# Facility management associated with improved primary health care outcomes in Ghana

**DOI:** 10.1371/journal.pone.0218662

**Published:** 2019-07-02

**Authors:** Erlyn K. Macarayan, Hannah L. Ratcliffe, Easmon Otupiri, Lisa R. Hirschhorn, Kate Miller, Stuart R. Lipsitz, Atul A. Gawande, Asaf Bitton

**Affiliations:** 1 Ariadne Labs, Brigham and Women’s Hospital & Harvard T.H. Chan School of Public Health, Boston, MA, United States of America; 2 Department of Global Health and Population, Harvard T.H. Chan School of Public Health, Boston, MA, United States of America; 3 Kwame Nkrumah University of Science and Technology, Kumasi, Ghana; 4 Feinberg School of Medicine, Northwestern University, Chicago, IL, United States of America; 5 Center for Surgery and Public Health, Brigham and Women’s Hospital, Harvard Medical School, Boston, MA, United States of America; 6 Department of Surgery, Brigham and Women’s Hospital, Boston, MA, United States of America; 7 Center for Primary Care, Harvard Medical School, Boston, MA, United States of America; 8 Department of Health Care Policy, Harvard Medical School, Boston, MA, United States of America; 9 Division of General Medicine, Brigham and Women’s Hospital, Boston, MA, United States of America; TNO, NETHERLANDS

## Abstract

**Background:**

Strong primary health care (PHC) is essential for achieving universal health coverage, but in many low- and middle-income countries (LMICs) PHC services are of poor quality. Facility management is hypothesized to be critical for improving PHC performance, but evidence about management performance and its associations with PHC in LMICs remains limited.

**Methods:**

We quantified management performance of PHC facilities in Ghana and assessed the experiences of women who sought care at sampled facilities. Using multi-level models, we examined associations of facility management with five process outcomes and eight experiential outcomes.

**Findings:**

On a scale of 0 to 1, the average overall management score in Ghana was 0·76 (IQR = 0·68–0·85). Facility management was significantly associated with one process outcome and three experiential outcomes. Controlling for facility characteristics, facilities with management scores at the 90^th^ percentile (management score = 0·90) had 22% more essential drugs compared to facilities with management scores at the 10^th^ percentile (0·60) (p = 0·002). Positive statistically non-significant associations were also seen with three additional process outcomes—integration of family planning services (p = 0·054), family planning types provided (p = 0·067), and essential equipment availability (p = 0·104). Compared to women who sought care at facilities with management scores at the 10^th^ percentile, women who sought care at facilities at the 90^th^ percentile reported 8% higher ratings of trust in providers (p = 0·028), 15% higher ratings of ease of following provider’s advice (p = 0·030), and 16% higher quality rating (p = 0·020). However, women who sought care in the 90^th^ percentile facilities rated their waiting times as worse (22% lower, p = 0·039).

**Interpretation:**

Higher management scores were associated with higher scores for some process and experiential outcomes. Large variations in management performance indicate the need to strengthen management practices to help realize the full potential of PHC in improving health outcomes.

## Introduction

In October 2018, the world celebrated the fortieth anniversary of the Alma Ata Declaration, which established primary health care (PHC) as the key mechanism for achieving the ambitious goal of “Health for All.”[1] The anniversary came at an auspicious time, with the global community turning its focus to achieving Universal Health Coverage (UHC).[2] While the centrality of PHC in this effort is recognized,[3] PHC services in many low- and middle-income countries (LMICs) are often of strikingly poor quality.[4] Furthermore, significant knowledge gaps remain on determinants of improved PHC facility performance in LMICs.[5] To achieve UHC and deliver on the promise of Alma Ata, more evidence is needed about which bottlenecks are most detrimental to PHC system performance and the best strategies to overcome these barriers.

Facility management is assumed to be an important contributor to PHC performance.[6] In high-income countries, substantial evidence exists that better management is associated with improved facility performance and health outcomes, particularly in hospitals.[7–9] Across nine mostly high-income countries, management practices in inpatient settings were strongly associated with better clinical outcomes including mortality rates from myocardial infarctions and surgeries, shorter waiting lists, and reduced staff turnover.[9] However, what constitutes “good” PHC management and its impact on performance is less known in LMICs. Available evidence comes almost exclusively from the hospital settings,[10] with little known about management of PHC facilities or management’s effect on PHC quality or patient experience. Further limiting our understanding of PHC facility management effects on care delivery in LMICs is the lack of validated measurement tools that can be implemented at scale. The best available evidence from both low- and high-income countries relies primarily on detailed case studies,[11] extensive 360° reviews of individual manager’s performance,[12] or in-depth qualitative interviews[13]–methods which generate rich data but are costly, time-consuming, and difficult to replicate at scale.

In this paper, we examined the associations of management on service delivery process outcomes and women’s experience of care using a new survey methodology for assessing management of PHC facilities. The survey was implemented in a national sample of facilities across Ghana, a middle-income country of 28 million people with an average life expectancy of 66 years,[14] to determine the level and variations in management performance by region and facility type. Ghana’s health system includes both private and public facilities. The public sector provides approximately 65% of care delivery,[15] with PHC services delivered through a range of facilities including Community-Based Health Planning and Services (CHPS) compounds, Ghana’s most basic, community-based PHC facility; Health Centers; and District Hospitals. District hospitals provide comprehensive health care and are responsible for partnering with the District Health Administration and local government to plan, supervise, monitor, and coordinate service delivery, while health centers are responsible for planning, developing, monitoring, and evaluating community-based service delivery. CHPS facilities work at the community level to provide promotive, preventive, and basic curative care through facility and home-based care.[16] To our knowledge, this is the first national survey to quantify management practices and explore its associations with PHC service quality in Sub-Saharan Africa.

## Methods

### Adaptation of a management framework

To identify a framework for measuring PHC facility management performance, we undertook an extensive scoping effort, the methods and results of which are described in the S1 File. We identified the World Management Survey (WMS) as a particularly well-validated and influential framework for measuring management performance across sectors.[13,17] The WMS identifies four domains of management: Operations, Performance Monitoring, Target Setting, and Human Resources.[17] We added a fifth domain—Community Engagement—that is essential for high-quality management of PHC facilities in LMICs.[11]

### Data sources

#### Survey design

The WMS employs an intensive, qualitative methodology that is difficult to implement at scale in LMIC PHC facilities, so we used our adapted WMS framework to guide the development of a new, quantitative, close-ended facility survey suitable for this setting. We also designed a household survey to assess women’s experience of PHC services in Ghana, drawing extensively from validated survey questions including measures of responsiveness from the WHO World Health Survey Responsiveness Module[18] and respectfulness of care and future care seeking intentions measures from work in Tanzania and Ethiopia.[19,20] The surveys were administered in 2016 in partnership with the Performance Monitoring and Accountability 2020 (PMA2020) platform and the Kwame Nkrumah University of Science and Technology (KNUST) through integration into existing PMA2020 facility and household surveys of women of reproductive age, designed to track progress towards family planning targets.[21] Surveys were administered in English, with translation into local languages as needed for the household survey. Because not all local languages in Ghana have a written form, hard copy translations of the surveys tools in all local languages were not developed, but enumerators with full English and local-language fluency undertook a structured, guided process to ensure consistency of verbal translations. English-language surveys are available in S2 File and S3 File.

#### Study sites

The Ghana Statistical Service selected 100 enumeration areas across Ghana’s ten regions with probability proportional to size using a master sampling frame stratified by urban-rural areas. All public health facilities that served each enumeration area and any private facilities (hospitals, polyclinics, and clinics) within its boundaries were included in the sample. If the sampled facility had inpatient services, questions related to service readiness and service delivery were confined to outpatient services only. In total, 142 facilities offering PHC services were surveyed with at least one facility representing each enumeration area and six to 25 PHC facilities per region. Within each of these same enumeration areas, 42 households were selected using a random number generator to complete the household survey. The household survey sample size was calculated to enable nationally-representative estimates of modern contraceptive prevalence rates within three percentage points.[21]

#### Facility survey administration

At each facility, trained interviewers asked to speak with the head of the facility. Eligible respondents included the Medical Director/Superintendent, Director of Nursing, or Nursing Matron at hospitals; Nurse, Midwife, Physician Assistant, or Physician In-Charge at health centers; and midwife or Community Health Nurse within CHPS facilities. At private facilities, eligible respondents included the owner, managing partner, administrator, and/or highest-ranking doctor. Respondents were allowed to refer the interviewer to additional facility staff to answer specialized questions, as needed. Facility-level data were collected from 19 September to 14 December 2016.

#### Household survey and linking to facility survey

All sampled households with at least one woman of reproductive age (aged 15–49 years) were included. All women of reproductive age residing in the household were identified, and efforts were made to interview all women who consented, including returning at different times of day to identify a time that respondents were available. Women who reported seeking care for themselves or a family member within the last six months were asked to identify which facility they went to for their most recent visit. If the facility was also included in our facility sample, the respondents and the facility surveys were matched, allowing us to make a direct link between women’s reports of their care experience and facility-level data. The household survey data were collected from 24 August to 23 November 2016, overlapping with facility-level data collection for three months.

### Variables

We assessed three categories of pre-specified outcomes, following STROBE guidelines (see S4 File): facility management scores, process outcomes at the facility level, and women-reported experiential outcomes:

#### Management

We categorized 27 indicators from the facility survey into the five management domains. All indicators were rescaled from 0 (lowest) to 1 (highest) (see S5 File). “Do not know” and missing responses for each indicator were treated as zero if it was determined by the research team that the respondent was responsible for knowing the answer.

#### Process outcomes: Facility level

Five process outcomes were selected based on their feasibility of assessment through the established survey platform and the data available through the core PMA2020 family planning survey. All outcomes were scaled from 0 (low) to 1 (high).

Essential Drug Index—proportion of availability of up to 21 drugs selected from the Service Delivery Indicators essential drugs list [22] adjusted for drugs expected to be available at each facility type according to the Ghana essential medicines list (see S6 File).[23] Information about drug availability was missing for five facilities which were excluded from the drug availability analysis.

1. Equipment Index—proportion of availability and functionality of six basic pieces of equipment (stethoscope, sphygmomanometer, child and adult weighing scale, thermometer, and any form of sterilization equipment), selected from the Service Delivery Indicators list of essential equipment.[22]
2. Integration of family planning services into maternal and child health (MCH) services and HIV services–If a facility offered both MCH and HIV services, a score of 1 was given if family planning was integrated in all these services. Otherwise, facilities were given a score of 0. Twenty-one facilities were excluded from this analysis for not offering MCH services (n = 16), HIV services (n = 1), or both (n = 4).
3. Index for family planning types provided—proportion of family planning types provided out of up to 13 types that should be available at each facility type based on national guidelines.[24] (see S7 File)
4. Index for family planning types counseled—proportion of family planning types counseled out of up to 16 types, including natural family planning methods, that should be available based on national guidelines.[24] (see S7 File)

#### Experiential outcomes: Woman respondent level

We assessed six outcomes related to responsiveness—as defined by the World Health Survey Responsiveness Module [18]—and two quality of care outcomes based on respondents’ ratings of their most recent experience seeking care for themselves or a family member within the last six months. All ratings were on a 5-point Likert scale and outcomes were scaled from 0 (low) to 1 (high).

1. Prompt attention to health needs–indicated by ratings for waiting times for consultation and treatment;
2. Basic amenities of health services–indicated by ratings of facility cleanliness;
3. Trust in the skills and abilities of facility health providers;
4. Dignity–indicated by ratings of level of respect shown by facility health providers;
5. Ease of understanding information from health provider;
6. Ease of following health advice of provider;
7. Likelihood of returning to the facility for future care;
8. Overall rating of quality of care received.

#### Control factors

Facility characteristics captured included: facility type; region; managing authority (public or private, including faith-based organizations); approval status to receive National Health Insurance Scheme (NHIS) reimbursements, a government social intervention program to provide financial access to health care; and facility size, defined by the number of beds. Household survey respondent sociodemographic information captured included age, educational attainment, marital status, insurance coverage, borrowing money or selling something to afford the cost of care, and region of residence.

### Statistical analysis

Using facility-level data, scores for each of the five management domains were calculated as unweighted averages of each set of indicators per domain, with scores ranging from 0 (lowest) to 1 (highest). Scores of all five management domains were averaged to calculate an overall management score. We measured reliability of the domain scores by computing Chronbach’s Alpha. Generalized linear models with a log link were used to model the facility-level process outcomes and individual-level experience outcomes as a linear function of overall management score (as a continuous covariate on the scale from 0 to 1) and all facility and/or individual-level control factors. A machine learning technique—supervised principal components[25]–was used to incorporate highly correlated predictors into the model to minimize confounding bias. Use of the log link allowed us to interpret the effect of management scores on each outcome as a ratio of outcome means (or proportions); for ease of interpretation, we display the ratio of adjusted outcome means for the 90^th^ percentile management score (= 0·90) versus the 10^th^ percentile management score (= 0·60). In the regression model with the log link, this ratio is estimated by multiplying the regression coefficient of continuous management score by the difference between the 90^th^ and 10^th^ percentiles of management score and then exponentiating this product. PMA2020 employs a complex survey design, utilizing survey weights, stratification by enumeration area, and clustering by service delivery points. [26] All analyses accounted for this complex survey design by adjusting for stratification, clustering, and weighting. Since the woman respondent-level analysis was restricted to include only those who sought care at a sampled facility, we adjusted the standard PMA2020 survey weights [26] by the inverse probability of seeking care. The probability of seeking care was estimated by fitting a logistic regression model that included the respondent and facility-level characteristics as covariates from our sample.[27] The facilities in our sample are an unweighted, stratified (by enumeration area), random sample of facilities in Ghana.[26] All analyses were conducted in Stata version 15·0 (StataCorp, LP, College Station, TX). AB, LRH, SRL, KM, EKM, and HLR had full access to the data. AB, EKM, and HLR had full responsibility for final submission of the manuscript.

### Ethics

All study participants provided informed, written consent. Non-literate respondents were requested to have a witness present to review the consent form, and the witness provided written consent alongside the respondent’s thumbprint. Participants under 18 years of age were consented alongside a parent or guardian.

This study was approved by School of Medical Sciences/Komfo Anokye Teaching Hospital Committee on Human Research Publications and Ethics (protocol CHRPE/AP/740/1.3), the Johns Hopkins School of Public Health Institutional Review Board (protocol 7238), and the Partners Human Research Committee (protocol 2016P002284).

### Role of the funding source

Funding was provided by the Bill & Melinda Gates Foundation. The funders played no role in study design; collection, analysis, and interpretation of data; writing of the paper; or decision to submit for publication.

## Results

One hundred and forty-two facilities providing PHC services were included in the management analysis (Table 1). Hospitals and polyclinics made up half of the sample (*n* = 71), followed by health centers/clinics (*n* = 48), and CHPS (*n* = 23). Of the 142 facilities, 16·2% were private and 97·2% were approved for reimbursement from the NHIS. The average number of beds per facility was 51 (SD = 63).

**Table 1:** Characteristics of facilities offering primary health care services and of women who sought primary health care services at a sampled facility.

Characteristics	Women who sought primary health care services at a sampled facility^»^	Facilities offering primary health care services
	(*n* = 896)	(*n* = 142)
**Region**	**Woman’s residence N (%)**	**Facility location N (%)**
Ashanti	153 (17·1)	25 (17·6)
Brong-Ahafo	101 (11·3)	13 (9·2)
Central	79 (8·8)	18 (12·7)
Eastern	96 (10·7)	19 (13·4)
Greater Accra	115 (12·8)	12 (8·5)
Northern	87 (9·7)	12 (8·5)
Upper East	33 (3·7)	6 (4·2)
Upper West	69 (7·7)	8 (5·6)
Volta	47 (5·2)	10 (7·0)
Western	116 (12.9)	19 (13·4)
	**Facility where care was sought:**	**Facility offering PHC:**
**Facility type**		
Hospitals/polyclinics	496 (55·4)	71 (50·0)
Health centers and clinics	249 (27·8)	48 (33·8)
CHPS	151 (16·9)	23 (16·2)
**Managing authority**		
Public	802 (89·5)	119 (83·8)
Private	94 (10·5)	23 (16·2)
**Facility size**	Mean = 59	Mean = 51
	Range (0–227)	Range (0–273)
Insurance*		
No	264 (29·5)	4 (2·8)
Yes	632 (70·5)	137 (97·2)
**Educational attainment**		
Never attended	172 (19·2)	
Primary	156 (17·4)	
Middle/JSS	359 (40·1)	
Secondary/JSS	148 (16·5)	
Higher	61 (6·8)	
**Marital status**		
Married	496 (55·4)	
Cohabitation	137 (15·3)	
Divorced/separated	61 (6·8)	
Widow	25 (2·8)	
Never in union	177 (19·7)	
**Residence**		
Rural	468 (52·2)	
Urban	428 (47·8)	
**Had to borrow money or sell something to afford visit costs**	200 (22.3)	
**Age**	Mean = 31	
	Range = 15–49	

Data given as number (percent) unless otherwise indicated.

*At the woman-respondent level, “yes” indicates the respondent had any form of insurance coverage. At the facility level, “yes” indicates that the facility is approved to receive National Health Insurance Scheme reimbursements. One facility had missing information on NHIS approval.

»Woman respondent-level data used the PMA2020 design survey weights (See Methods).

**Fig 1:**
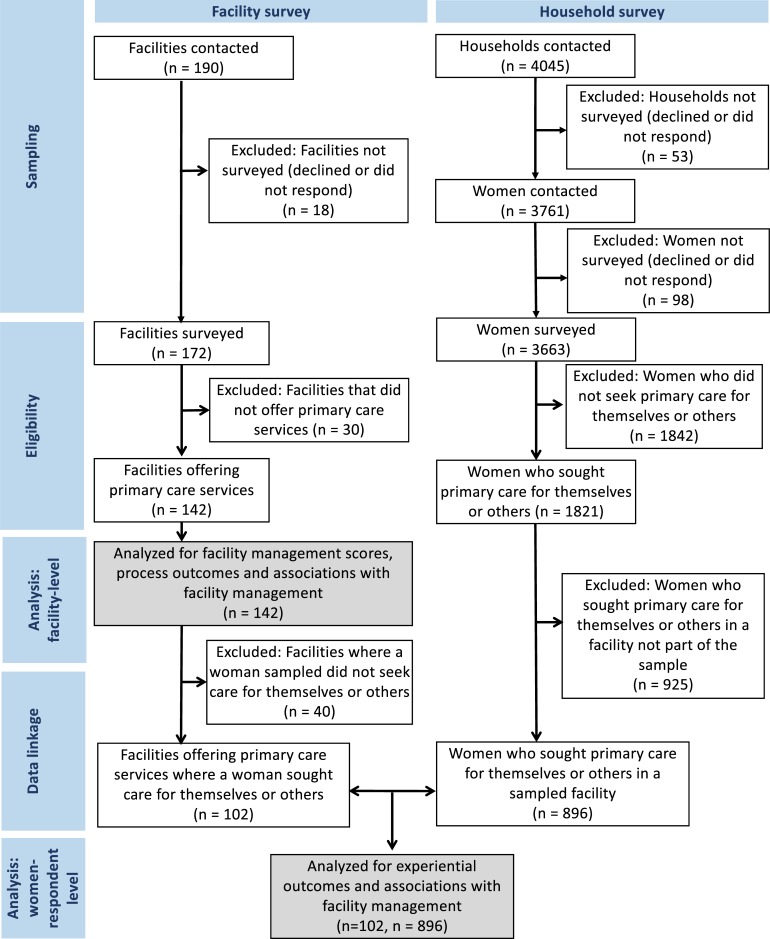
Analysis and linking of facility- and woman respondent-level datasets.

Abbreviations: CHPS Community-based Health Planning and Services; JSS Junior Secondary School. In total, 1821 (49·7%) of the 3663 women interviewed sought PHC services for themselves or family members in the last six months, with 896 (49·2%) seeking care from one of the sampled facilities (Fig 1). We found no significant differences in education or wealth quintile between women who sought care in a sampled facility versus another facility. Hospitals and polyclinics were the most frequent level of facility at which women sought care (55·4%), followed by health centers and clinics (27·8%), and CHPS (16·9%). The majority (89·5%) of women sought care in public facilities, and 70·5% reported having some form of insurance coverage. Nearly one-quarter (22·3%) of women reported having to borrow money or sell something to afford the cost of the visit.

The Chronbach’s Alpha values for each management domain (target setting, operations, human resources, monitoring, and community engagement) were 0.06, 0.56, 0.61, 0.85, and 0.66, respectively. The average overall management score was 0·76 (SD = 0·12) (Table 2), with significant variation across management domains. Human Resources was the highest scoring domain (mean = 0·89; SD = 0·17), while Community Engagement (mean = 0·65, SD = 0·20) was lowest.

**Table 2:** Indicators of management performance and average scores of all facilities per management domain.

Management variables (*n* = 142)	Mean (SD)
**Overall Management score**	**0**·7**6 (0**·**12)**
**Average Target Setting score**	**0**·**74 (0**·**25)**
Measures coverage of key population indicators such as immunization coverage	0·92 (0·28)
Has one comprehensive annual budget for running costs	0·71 (0·45)
Reports accountability for health outcomes of a group of people	0·59 (0·49)
**Average Operations score**	**0**·**73 (0**·**18**)
Has hand washing area with soap and water available^	0·95 (0·22)
Has healthcare worker present in the facility 24 hours a day	0·92 (0·28)
Open every day	0·85 (0·36)
Facility head has received any formal management training	0·76 (0·43)
Has user fees displayed	0·45 (0·50)
Proportion of time facility head spent on managerial activities the previous day~	0·43 (0·24)
**Average Human Resources score**	**0**·**89 (0**·**17)**
Staff are offered training to improve their skills	0·99 (0·12)
Supervisors have held individual meetings to review staff performance	0·95 (0·22)
Has established criteria to evaluate staff performance	0·82 (0·38)
Has formal, supportive, and continuous supervision system+	0·79 (0·29)
**Average Monitoring score**	**0**·**81 (0**·**15)**
Maintains books to track revenue and expenditure	0·97 (0·17)
Conducts quality improvement activities	0·94 (0·24)
Held meetings to discuss routine service statistics with staff or clinical audit data	0·94 (0·23)
Has mechanism to report new disease outbreaks	0·93 (0·26)
Extent to which data to monitor and improve service delivery is valued at the facility*	0·88 (0·19)
Tracks common conditions	0·88 (0·33)
Reports client opinions using any available tool	0·54 (0·50)
Regularly receives reports tracking common conditions with results shared with staff	0·41 (0·21)
**Average Community Engagement score**	**0**·**65 (0**·**20)**
Collects client opinions using any tool	0·95 (0·22)
Shared information on performance with the community in the past 12 months	0·78 (0·41)
Patients’ opinions drive change or improvement from rare (0) to very often (1) *	0·67 (0·20)
Has made changes based on client opinion in the last six months	0·64 (0·48)
Has a community advisory board that meets regularly and follows up	0·52 (0·49)
Has a community member regularly attending staff meetings	0·31 (0·46)

Overall management and domain scores are averages of component indicators on a scale of 0 (lowest) to 1 (highest). All component indicators are dichotomous from 0 (no or “do not know”) to 1 (Yes) unless otherwise indicated.

^ Ranges from no handwashing area (0), with handwashing area but no soap and water (0.33), with handwashing area and either soap or water (0.66), and with handwashing area, soap and water (1).

~ Continuous indicator

+ No method of supervision (0); Formal supervision process with regular pre-arranged supervision meetings (0.33); Supervision is available if requested by staff or supervision consists of negative feedback when performance is poor (0.66); Supervision is supportive and continuous (1)

* Five-point Likert scales from 0 to 1 (increments of 0.

**Fig 2:**
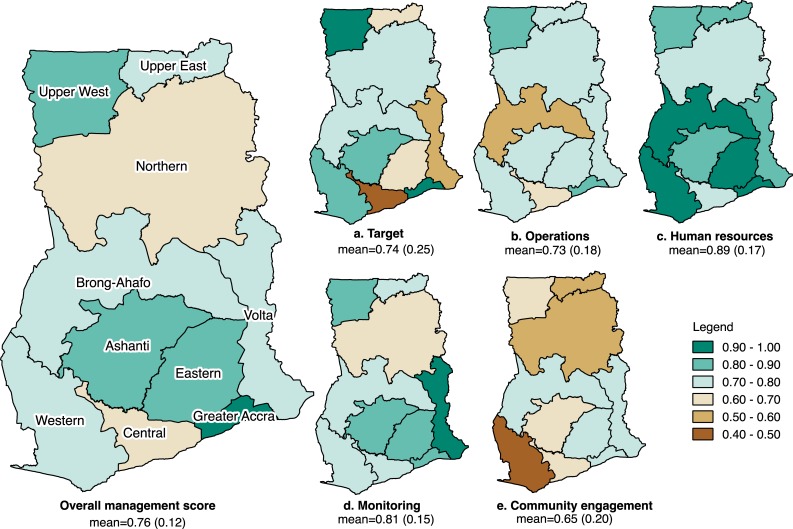
Regional variations in overall management and for each management domain in Ghana (n = 142). Regions are colored based on average management scores (in absolute values) with the lowest regional score in brown to the highest regional score in green. Values shown below each map are the national averages and standard deviations.

Regional disparities existed in overall management performance and specific domain performance (Fig 2); facilities in the Central region scored lowest (mean = 0·64, SD = 0·10) and facilities in Greater Accra region scored highest (mean = 0·90, SD = 0·05) in most domains.

**Fig 3:**
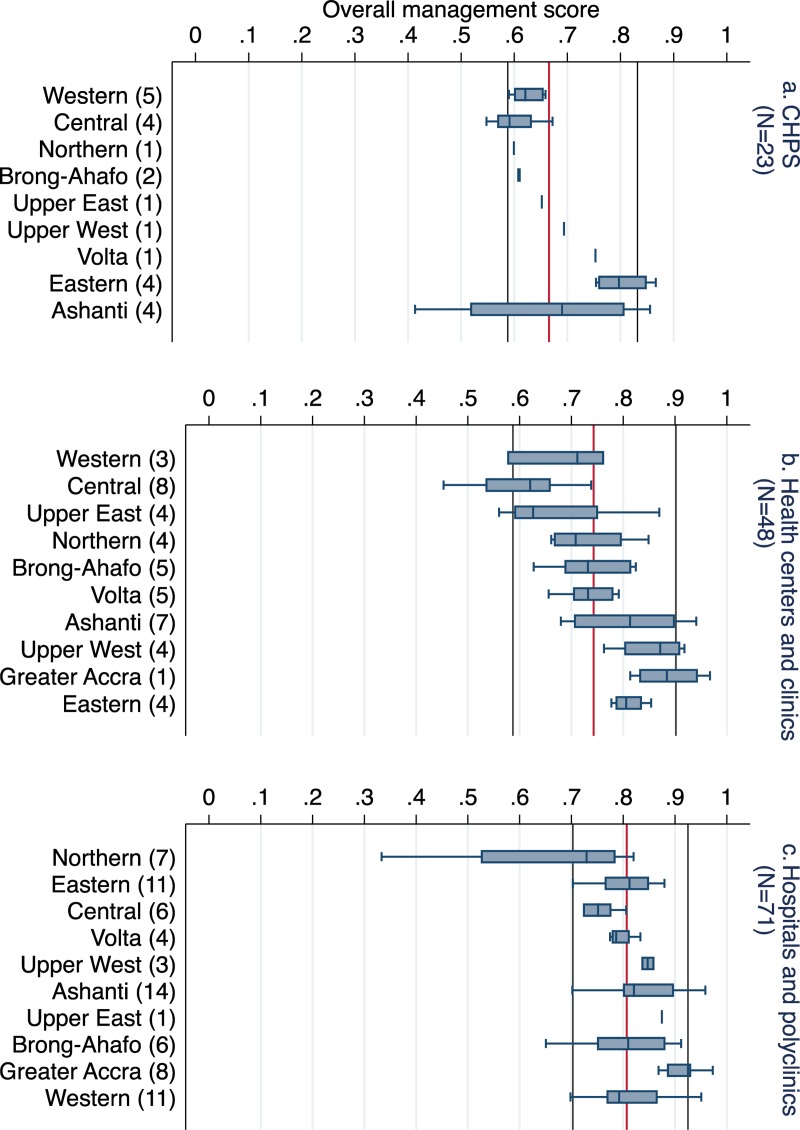
Differences in overall management of primary care facilities in Ghana by facility type and region (n = 142). Box plots show the median line by facility types: Community-based Health Planning and Services (CHPS) (mean = 0·67), health centers and clinics (0·74), and hospitals and polyclinics (0·81). Numbers beside each region name (in parentheses) refer to the number of sampled facilities per region. CHPS facilities in Greater Accra were not represented in the sample. The outer box plot edges span the 25th to 75th percentile, and whiskers represent the 95^th^ percentiles. The lines in the y-axes represent the 10th and 90th percentiles of management scores (blue) and the mean of overall management score (red) for each type of facility.

Management performance varied significantly by facility type (p<0·0001), with hospitals and polyclinics performing better overall than CHPS and health centers/clinics, whose performance was more variable (Fig 3). Additionally, significant variations existed by facility type in performance of individual management domains (see S8 File).

We also found significant differences in process and experiential outcomes by region and facility type. The average essential drug index was 0·74, ranging from 0·60 in the Northern and Upper East regions to 0·88 in Greater Accra (Table 3 and S9 File). Hospitals/polyclinics had the highest average essential drug index (mean = 0·88) compared to health centers/clinics and CHPS. The majority of all facilities across all regions had a high equipment index (mean = 0·97). Although family planning integration into maternal and HIV services was high (mean = 0·88), on average, facilities offered only 59% of family planning types included in national policies and counseled on only 73% of methods. CHPS had the highest scores for family planning types counseled (mean = 0·75) but the lowest scores for family planning types provided (mean = 0·50).

**Table 3:** Differences in essential supplies and women respondent’s experience of care in Ghana by region and facility type.

			Regions											Type of facility			
	Overall mean	Overall SD	Ashanti	Brong-Ahafo	Central	Eastern	Greater Accra	Northern	Upper East	Upper West	Volta	Western	P values*	Hospitals and poly-clinics	Health centers and clinics	CHPS	P values*
**A. Process outcomes**																	
1. Essential drug index^+^	0·74	0·21	0·76	0·78	0·69	0·72	0·88	0·60	0·60	0·76	0·74	0·79	0·19	0·88	0·67	0·50	<0·0001
2. Equipment index	0·97	0·07	0·96	0·99	0·97	0·97	0·99	0·97	0·94	0·98	0·95	0·98	0·99	1·00	0·96	0·92	0·0300
3. FP integration~	0·88	0·33	0·91	1·00	0·79	0·88	1·00	0·73	0·67	1·00	0·86	0·87	0·95	0·90	0·95	0·54	0·08
4. FP types provided	0·59	0·20	0·65	0·60	0·47	0·57	0·65	0·58	0·50	0·59	0·60	0·61	0·08	0·60	0·61	0·50	<0·0001
5. FP types counseled	0·72	0·20	0·79	0·75	0·61	0·78	0·79	0·71	0·49	0·67	0·63	0·80	<0·0001	0·73	0·70	0·75	0.0080
**B. Experiential outcomes**^																
6. Prompt attention (waiting time)	0·65	0·35	0·63	0·68	0·64	0·69	0·60	0·60	0·74	0·59	0·76	0·68	0·21	0·59	0·72	0·76	<0·0001
Waiting time (in mins)	9·32	12·68	8·79	7·59	9·58	8·90	11·32	15·52	7·65	4·81	11·22	8·23	<0·0001	8·96	8·98	11·91	0·005
7. Facility cleanliness	0·60	0·20	0·61	0·63	0·59	0·55	0·67	0·56	0·57	0·49	0·67	0·57	<0·0001	0·59	0·60	0·61	0·58
8. Trust in providers	0·70	0·16	0·68	0·71	0·75	0·69	0·77	0·69	0·68	0·57	0·76	0·68	0·024	0·72	0·66	0·72	0·007
9. Respectfulness	0·57	0·21	0·61	0·55	0·65	0·53	0·63	0·54	0·50	0·51	0·63	0·52	<0·0001	0·57	0·56	0·61	0·35
10. Ease of understanding information from providers	0·69	0·16	0·68	0·69	0·80	0.67	0·74	0·70	0·75	0·57	0·67	0·70	<0·0001	0·70	0·66	0·76	0·001
11. Ease of following advice from providers	0·71	0·17	0·70	0·71	0·77	0·69	0·77	0·69	0·77	0·62	0·68	0·69	0·003	0·71	0·68	0·76	0·015
12. Likelihood of returning to the same facility	0·70	0·18	0·71	0·69	0·83	0·71	0·65	0·76	0·68	0·66	0·67	0·68	<0·0001	0·70	0·68	0·74	0·024
13. Overall quality of care	0·61	0·21	0·66	0·65	0·66	0·56	0·66	0·57	0·53	0·51	0·64	0·56	<0·0001	0·62	0·60	0·63	0·26

Unless otherwise noted, n = 142 facilities for process outcomes and n = 896 respondents for experiential outcomes.

* Cells are shaded if statistically significant at p <0·05. P values were adjusted for multiple comparison testing using Bonferroni correction. Adjustments were done among process outcomes and among experiential outcomes.

+ N = 137 (excludes 5 facilities with missing drug information)

~ N = 121 (excludes 21 facilities that did not offer both maternal and child health (MCH) and HIV services, including four facilities that offered neither, one that did not offer HIV services, and 16 that did not offer MCH services)

^ Experiential outcome measures are scaled from 0 to 1.

The average waiting time reported was 9·32 minutes, and the average acceptability rating of these waiting times was 0·65. Ratings of wait times were significantly better at CHPS facilities than hospitals/polyclinics (mean = 0·76 versus mean = 0·59, p<0·0001) despite slightly higher average wait times (11·91 minutes versus 8·96 minutes). The average rating of facility cleanliness was 0·60, with substantial regional variation (p<0·0001) but little variation between facility types. Ratings for trust in providers were significantly higher at both CHPS and hospitals/polyclinics (mean = 0·72) than health centers/clinics (mean = 0·66) (p = 0·024). Among all experiential outcomes assessed, respect shown by providers had the lowest overall average (mean = 0·57), with ratings highest in CHPS. Ratings for both ease of understanding and following provider’s advice were moderately high (0·69 and 0·71, respectively), and significantly higher at CHPS facilities than other facility types (p = 0·001, p = 0·015) with significant regional variation (p<0·001, p = 0·003). Similarly, reported likelihood of returning to the facility was high at 0·70, with significantly higher ratings in CHPS facilities (p = 0·024) and significant regional variation (p<0·001). Overall quality of care ratings were moderate (0·61), with significant regional variation (p<0·0001) but little variation between facility types.

Controlling for facility characteristics, facilities with management scores at the 90^th^ percentile (management score = 0·90) had 22% more essential drugs compared to facilities with management scores at the 10^th^ percentile (management score = 0·60) (p = 0·002) (Table 4). Although we found no statistically significant differences for other process outcomes, positive associations occurred with three additional process outcomes—integration of family planning services (p = 0·054), family planning types provided (p = 0·067), and essential equipment availability (p = 0·104). Controlling for facility characteristics and women’s sociodemographic characteristics, women who sought care at facilities at the 90^th^ versus the 10^th^ percentile of management scores reported 8% higher ratings of trust in providers (p = 0·028), 15% higher ratings of ease of following provider’s advice (p = 0·030), and 16% higher overall quality rating (p = 0·020). Additionally, women in the 90^th^ versus 10^th^ percentile facilities rated their waiting times 22% lower (p = 0·039).

**Table 4:** Models of management performance, essential supplies and women respondent experience of care in Ghana.

		90th adjusted means	10^th^ adjusted means	Ratio of adjusted mean/proportion for 90^th^ vs 10^th^ percentile in Overall Management Score*	P value
**A. Process outcomes**					
1	Essential drug index^+^	0·80	0·66	1·22 (1·07–1·37)	0·002
2	Equipment index	0·99	0·95	1·04 (0·99–1·08)	0·104
3	Family planning integration^~^	0·93	0·78	1·19 (1·00–1·42)	0·054
4	Family planning types provided	0·62	0·53	1·16 (0·99–1·37)	0·067
5	Family planning types counseled	0·71	0·74	0·96 (0·84–1·09)	0·508
**B. Experiential outcomes^**					
6	**Prompt attention (waiting time)**	**0·58**	**0·75**	**0·78 (0·61–0·99)**	**0·039**
7	Facility cleanliness	0·65	0·59	1·10 (0·96–1·25)	0·164
8	**Trust in providers**	**0·79**	**0·73**	**1·08 (1·01–1·16)**	**0·028**
9	Respect rating	0·56	0·53	1·05 (0·90–1·22)	0·566
10	Ease of understanding provider’s advice	0·72	0·70	1·03 (0·95–1·11)	
11	**Ease of following provider’s advice**	**0·79**	**0·69**	**1·15 (1·01–1·30)**	**0·030**
12	Likelihood of returning to the facility	0·73	0·73	1·00 (0·93–1·09)	0·919
13	**Quality rating**	**0·72**	**0·62**	**1·16 (1·02–1·32)**	**0·020**

Unless otherwise stated, n = 142 for process outcomes and n = 896 for experiential outcomes. Cells are shaded if statistically significant at p <0·05.

+N = 137 (excludes 5 facilities with missing drug information)

~ N = 121 (excludes 21 facilities that did not offer both maternal and child health (MCH) and HIV services, including four facilities that offered neither, one that did not offer HIV services, and 16 that did not offer MCH services.)

^ Experiential outcome measures are scaled from 0 to 1.

* Generalized linear models with a log link were used for each outcome, with management score as a continuous covariate (on scale from 0 to 1). We display the ratio of adjusted means/proportions for the 90^th^ versus 10^th^ percentiles in management score. In the regression model with the log link, this ratio is estimated by multiplying the regression coefficient of continuous management score by the difference between the 90^th^ and 10^th^ percentiles, and then exponentiating this product. Supervised principal components^24^ accounted for the following control variables: A) facility type, region, managing authority, whether the facility was approved to receive National Health Insurance Scheme reimbursements; and facility size, defined by the number of beds; B) all accounted for under A, plus respondent’s age, educational attainment, marital status, insurance coverage, borrowing money or selling something to afford the cost of care, and region of residence.

## Discussion

This was the first known national study to quantify management performance in PHC facilities in an LMIC and associate it with process and experiential outcomes. Higher facility management scores were independently associated with higher essential drug availability and higher ratings in four of the eight experiential outcomes assessed.

Elements of experiential quality which were higher in better-managed facilities included promptness of care, issues related to provider-client interactions (trust, communication), as well as overall user rating of quality. Trust and overall perceived quality of care have been shown to be important predictors of care seeking behavior and bypass of health facilities for maternal and child health services, indicating that management is an important improvement target.[28] CHPS facilities had higher ratings on most experiential outcomes, potentially reflecting their strong focus on community engagement,[29] but lower scores on most process outcomes. Significant variability in experiential quality was seen across regions, with some regions tending to score lower across multiple domains of experiential quality. Together, these findings indicate that more a more systemic focus on improving experiential quality—rather than facility-by-facility efforts—is needed to ensure access to high-quality experiential care across all regions and facility types.

Our finding that better-managed facilities have higher essential drug availability is consistent with other available evidence. For example, Mabuchi found key PHC facility characteristics associated with better performance in a Performance-Based Financing scheme in Nigeria that are closely aligned with the management categories described here, including setting targets, monitoring progress towards targets, and strong community engagement.[11] A randomized-controlled trial of 80 PHC facilities in Nigeria found that a management consulting program led to improvements in outcomes such as supply availability and facility cleanliness, though these changes were not sustained one year later.[30]

We found significant differences in management performance across management domains, regions, and facility types in Ghana. The Human Resources domain was nearly uniformly high-scoring, while Community Engagement performance was low overall and highly variable across regions. Higher-level facilities had better management than lower-level facilities, though for each type of the facility the performance spectrum was wide and overlapping. Greater Accra region was the highest-performing region, while Northern and Central regions were the lowest, however all regions performed variably across management domains and each had a different performance profile. Together, these findings indicate that gaps in management performance are not confined to specific domains, regions, or facilities and highlight the need to improve facility management across all facility types and in CHPS facilities in particular, given the central role of CHPS in PHC service delivery.[16]

However, there is relatively limited evidence about which improvement strategies are best suited to the PHC context. In Ethiopia, a multipronged initiative aimed at improving management practices by strengthening management personnel through practice-based training, on-site mentorship and a Master of Hospital Administration resulted in improvements in 86 hospital performance standards from 27% at baseline to 51%.[31] Further work is needed to understand whether interventions effective at the hospital level in Ethiopia will also strengthen management performance at the PHC level and result in improvements in facility readiness, patient experience, or health outcomes. Evidence also suggests that management practices at the district level are key determinants of performance [32] and that management of PHC facilities may be most important in settings where PHC facility managers have at least a baseline level of autonomy to enact their agendas.[11] The regional variation documented in this study highlights the need to understand the broader subnational systems and context which may influence management culture and effectiveness at the facility level.

Our study had several limitations. Although the facility survey was a stratified, random sample of facilities, estimates from 2014 show that approximately 35% of health care services are provided in the private sector in Ghana,[15] suggesting that private sector facilities may be under-represented in our sample. However, we found no statistical difference in management scores between sampled private and public-sector facilities. Further, we were unable to assess how financial commitments from governmental and non-governmental sources or variable human resource capacities affect management performance; these areas should be the focus of future exploration. Additionally, our selection of facility process outcomes was limited by the data and respondents available through the PMA2020 survey platform and did not include measures of technical quality, assess the experiences of users other than women of reproductive age, or externally validate reported management capacity. The survey also did not capture women’s expectations of care, which may have confounded their reported experiences. Additionally, while we conducted pre-testing of the facility survey in multiple facilities in Ghana to ensure feasibility and acceptability, it was not previously formally validated for use in PHC settings in LMICs. The reliability scores of the management domains reported here indicate that improvements to the tool will be necessary. Further work is underway to repeat a modified version of this survey in Ghana and other LMIC contexts to assess the generalizability and reliability of the survey, including measuring experience in populations other than women of reproductive age.

## Conclusions

Our results suggest that higher PHC facility management scores are significantly and independently associated with essential drug availability as well as overall quality and components of responsiveness of care as reported by patients and families. The results have important implications for Ghana and the broader research community. For Ghana, the significant variations in performance across region and facility type highlight that a one-size-fits-all improvement approach is unlikely to succeed and that improvements will need to be targeted to the specific context and performance profile in question. Further work is also needed to examine how existing policies, governance systems, and national and sub-national quality infrastructure may affect facility management, and how this management impacts health outcomes over time. At the global level, our results are part of a growing body of evidence highlighting the need for increased research and policy to better measure key service delivery functions, including facility management, to inform improvement work critical to the achievement of quality PHC necessary for effective UHC.[2]

## Supporting information

S1 File Additional methodological reports. Additional information about the selection and modification of a management framework and model equations for the survey-weighted generalized linear models employed in this analysis.
(PDF)

S2 File Facility survey. Complete survey used to collect data at health facilities.
(PDF)

S3 File Household survey—female questionnaire. Complete survey used to collect data from individual women in their households.
(PDF)

S4 File STROBE checklist. (PDF)

S5 File Management component indicators based on the World Management Survey framework. Details of the 27 indicators which comprise the management index.
(PDF)

S6 File Essential drugs assessed. List of essential drugs assessed by facility type.
(PDF)

S7 File Family planning types assessed. List of family planning types assessed by facility type.
(PDF)

S8 File Management domains by region and facility type. Figure showing variation in performance of management overall as well as each management domain, by region and facility type.
(PDF)

S9 File Process outcomes by region. Maps showing regional variation across Ghana in process outcomes.
(PDF)

## Abbreviations

CHPS: Community-Based Health Planning and Services
FP: Family Planning
HIV: Human Immunodeficiency Virus
KNUST: Kwame Nkrumah University of Science and Technology
LMICs: Low- and Middle-Income Countries
MCH: Maternal and Child Health
NHIS: National Health Insurance Scheme
PHC: Primary Health Care
PMA2020: Performance Monitoring and Accountability 2020
SD: Standard Deviations
UHC: Universal Health Coverage
WMS: World Management Survey
