# Supplementary material for: Facility management associated with improved primary health care outcomes in Ghana
Source: PLoS One. 2019 Jul 2;14(7):e0218662. doi: 10.1371/journal.pone.0218662 (PMC6605853; doi:10.1371/journal.pone.0218662)
Supplement: S1 File — Additional information about the selection and modification of a management framework and model equations for the survey-weighted generalized linear models employed in this analysis. (PDF) [file pone.0218662.s001.pdf]

Supplementary Information 1: Additional Methodological Reports

Selection and Modification of a Management Framework

To begin the process of designing new management surveys, the authors conducted a targeted literature review of organization and management in LMICs along with interviews with experts in this arena, including:

1. Raffaella Sadun, Harvard Business School and World Management Survey
2. Alex Haynes, Ariadne Labs and World Management Survey
3. Mario Macis, Johns Hopkins University, IZA, and National Bureau of Economic Research (NBER)
4. Christophe Rockmore, Service Delivery Indicators (SDI), World Bank
5. Ezequiel Molina, SDI, World Bank
6. Shannon Barkley, PHC Service Delivery and Safety, WHO
7. Erin Sullivan, Harvard Medical School Center for Primary Care
8. Benjamin Loevinsohn, SDI, World Bank
9. Nittita Prasopa-Plazier, Patients for Patient Safety, WHO
10. Giorgio Cometto, Global Health Workforce Alliance, WHO
11. Nuria Toro-Polanco, Integrated People-Centered Health Systems, WHO
12. Kavitha Viswanathan, Health Statistics and Information Systems/Health Facility Harmonization, WHO
13. Shamsuzzoha Syed, Service Delivery and Safety, WHO
14. Hernan Montenegro, Service Delivery and Safety, WHO
15. Herbie Duber, Institute for Health Metrics and Evaluation
16. Erika Linnander, Yale University

Based on our review of the literature and recommendations from consulted experts, authors reviewed the following survey and measurement compilation sources for Facility Management and Organization survey questions and/or frameworks:

- Service Provision Assessment (SPA)[1]
- Service Availability and Readiness Assessment (SARA)[2]
- SafeCare[3]
- Patient Centered Medical Home-Assessment Tool[4]
- Primary Care Assessment Tools (PCAT)[5]
- Primary Care Assessment Survey (PCAS)[6]
- Commonwealth Fund Scorecard[7]
- World Bank and WHO UHC Monitoring Framework[8]
- WHO Handbook on Monitoring and Evaluation of Human Resources for Health[9]
- Canadian Institute for Health Information, Measuring Organizational Attributes of Primary Health Care Survey[10]
- USAID/Management Sciences for Health Leadership Development Program[11,12]
- Centers for Disease Control and Prevention, Sustainable Management Development Program[13–16]
- WHO Hospital Management Guidelines, 1998[17]
- Mohd-Shamsudin, 2012[18]
- Kitreeawutiwong, 2015[19]
- Munyewende, 2016[20]
- Rowe, 2010[21]
- Ethiopian Hospital Management Initiative[22–27]

One particularly important source of information was the World Management Survey (WMS).[28–33] The WMS is an innovative research project aimed at measuring the adoption of basic managerial practices. Started in 2001, the initiative has collected in-depth information on management processes across more than 15,000 organizations in manufacturing, retail, education and healthcare in 30 countries. Over the past 5 years, the WMS has conducted more than 1,800 interviews across acute care hospitals in the US, Europe, Asia and South America. Interviewers obtain and conduct interviews that evaluate and score management practices by defining the concept of "good" and "bad" management practices and codifying them

from 1 (worst practice) to 5 (best practice) across key management practices used by organizations across different sectors. These practices are grouped into four areas: 1) Operations Management, 2) Performance Monitoring, 3) Target Setting, 4) Talent Management. (In some WMS formats, a fifth domain: Leadership Management is also added.) Interviews last approximately 90 minutes and are conducted over the phone by extensively trained interviewers. However, given the technical expertise and extensive time commitment needed to conduct, score, and synthesize interviews, the authors determined that using the WMS in its current form would not be feasible.

The authors also met with Dr. Mario Macis of Johns Hopkins University, IZA, and NBER to discuss further options for survey adaptation. Dr. Macis has been involved in the evaluation of a program aimed at implementing SafeCare practices across primary health facilities in Nigeria.[34] SafeCare is an International Society for Quality in Healthcare (ISQua)-accredited set of standards that are specifically designed for low and middle-income contexts. SafeCare standards cover both clinical services and management functions and are designed to set up a step-wise improvement trajectory for facilities. SafeCare standards were informed by WMS practices but are in a more quantifiable and easily implementable survey format. However, the SafeCare evaluation tool includes more than 800 indicators, making it difficult for a small subset to reliably and fully measure relevant concepts.

Despite these difficulties in adapting World Management Survey questions for facility assessments in LMICs, the authors identified the WMS framework as a valuable tool for structuring a survey module for measuring management of PHC facilities in LMIC. Therefore, building on the literature and surveys reviewed, we modified the WMS framework to more directly apply to PHC facilities in LMIC. Our framework retains the core domains of WMS, including Target Setting, Performance Monitoring, Operations Management, and Human Resource Management. Additionally, we propose that a fifth domain—Community Engagement—is essential for high-quality management *particularly* of PHC facilities in the LMIC context.

Based on this management framework, the indicators listed in Supplemental Information 3 were included in the first round of PMA2020 survey data collection in Ghana in the fall of 2016. Wherever possible, indicators from validated surveys such as SPA and SDI were prioritized.

Facility and household survey modules were field tested in June 2016 as a component of the training of 20 Data Supervisors. Training and field-testing were overseen by PMA2020 staff from the Bill & Melinda Gates Institute for Population and Reproductive Health of the Johns Hopkins Bloomberg School of Public Health led the training, with support from Kwame Nkrumah University of Science & Technology (KNUST) School of Medicine project staff and the Ariadne Labs Primary Care Team. The facility module was field tested at a public District Hospital, a private polyclinic, and a CHPS compound in the areas surrounding Kumasi, Ghana. None of the facilities surveyed as part of field testing were in the survey sample. Facility survey field testing led to several modifications to the survey to improve clarity. Household surveys were field tested in five households in peri-urban areas surrounding Kumasi, Ghana. Both the survey and household field testing indicated that the surveys were feasible to implement and acceptable to both data collectors and respondents.

The list of management indicators was updated for the second round of data collection, which was conducted during the fall of 2017 and will be further modified for fielding in additional LMIC in late 2018.

Model Equations

In all our analyses, we used survey-weighted generalized linear models with a log link, i.e., we model the log of the means as a function of covariates:

$$\log[E(Y_i|x_i, \beta_i)] = \beta_0 + \beta_1 * x_{1i} + \beta_2 * x_{2i} \ldots + b_{0i}$$

or equivalently:

$$E(Y_i|x_i, \beta_i) = \exp(\beta_0 + \beta_1 * x_{1i} + \beta_2 * x_{2i} \ldots + b_{0i})$$

where $E(Y_i|x_i, \beta_i)$ is the mean of the particular outcome variable given the covariates.

For continuous or ordered (as in a Likert scale) Y_i, we estimated the betas using a non-linear least squares algorithm, which gives unbiased estimates regardless of the underlying distribution of the Y_i (the non-linear least squares algorithm does not require normal Y_i or homogeneity of variance to obtain unbiased estimate of the betas). The exponentiated regression

coefficients can be interpreted as a relative change (increase or decrease) in the mean of the outcome for a one unit increase in a covariate.

For dichotomous y (assuming it is coded 0 or 1), $E(Y_i|x_i, \beta_i) = prob(Y_i = 1|x_i, \beta_i)$, i.e., we are modelling the log of probability that the outcome = 1. For dichotomous y, we estimated the betas using maximum likelihood for binary data. The exponentiated regression coefficients for the log link for binary outcomes can be directly interpreted as the relative risk (ratio of probabilities that Y_i=1) for a one unit increase in a covariate. Although logistic regression is used more often for dichotomous outcomes, it produces odds ratios, and the odds ratio does not have as nice of an interpretation as the relative risk (for rare events, the OR and RR are similar, but the dichotomous outcomes in our study are not always rare, so we used the log link to directly obtain RRs).

References

1 The DHS Program - Service Provision Assessments (SPA). https://dhsprogram.com/What-We-Do/Survey-Types/SPA.cfm (accessed Jan 21, 2018).

2 WHO | Service Availability and Readiness Assessment (SARA). *WHO* 2015.

3 Safecare: Basic HealthCare Standards. http://www.safe-care.org/ (accessed Jan 21, 2018).

4 PATIENT-CENTERED MEDICAL HOME ASSESSMENT (PCMH-A) Introduction To The PCMH-A. .

5 Primary Care Assessment Tools. https://www.jhsph.edu/research/centers-and-institutes/johns-hopkins-primary-care-policy-center/pca_tools.html (accessed Jan 21, 2018).

6 Safran DG, Kosinski M, Tarlov AR, *et al.* The Primary Care Assessment Survey: Tests of Data Quality and Measurement Performance. *Med Care* 1998; **36**: 728–39.

7 Health System Scorecards - The Commonwealth Fund. http://www.commonwealthfund.org/publications/health-system-scorecards (accessed Jan 21, 2018).

8 Monitoring progress towards universal health coverage at country and global levels. 2014.

9 Handbook on Monitoring and Evaluation Human Resources for Health: with special applications for low-and middle-income countries Handbook on Monitoring and Evaluation of Human Resources for Health. 2009.

10 Measuring Organizational Attributes of Primary Health Care Survey. https://www.cihi.ca/sites/default/files/info_phc_organize_en.pdf (accessed Jan 21, 2018).

11 Kwamie A, van Dijk H, Agyepong IA. Advancing the application of systems thinking in health: realist evaluation of the Leadership Development Programme for district manager decision-making in Ghana. *Heal Res policy Syst* 2014; **12**: 29.

12 Seims LRK, Alegre JC, Murei L, *et al.* Strengthening management and leadership practices to increase health-service delivery in Kenya: an evidence-based approach. *Hum Resour Health* 2012; **10**: 25.

13 Centers for Disease Control. Sustainable Management Development Program: Two Decades of Improving Health Outcomes Through Strong Leadership and Management. 2002 DOI:10.1146/annurev.ecolsys.33.010802.150507.

14 Umble KE, Brooks J, Lowman A, *et al.* Management training in Vietnam's National Tuberculosis Program: an impact evaluation. *Int J Tuberc Lung Dis* 2009; **13**: 238–46.

15 Sucaldito NL, Tayag EA, Roces MCR, Malison MD, Robie BD, Howze EH. The Philippines Field Management Training Program (FMTP): strengthening management capacity in a decentralized public health system. *Int J Public Health* 2014; **59**: 897–903.

16 McEwan E, Conway MJ, Bull DL, Malison MD. Developing Public Health Management Training Capacity in Nicaragua. *Am J Public Health* 2001; **91**: 1586–8.

17 Conn CP, Jenkins P, Touray SO. Strengthening health management: Experience of district health teams in The Gambia. *Health Policy Plan* 1996; **11**: 64–71.

18 Mohd-Shamsudin F, Chuttipattana N. Determinants of managerial competencies for primary care managers in Southern Thailand. *J Health Organ Manag* 2012; **26**: 258–80.

19 Kitreerawutiwong K, Sriruecha C, Laohasiriwong W. Development of the competency scale for primary care managers in Thailand: Scale development. *BMC Fam Pract* 2015; **16**: 174.

20 Munyewende PO, Levin J, Rispel LC. An evaluation of the competencies of primary health care clinic nursing managers in two South African provinces. *Glob Health Action* 2016; **9**: 32486.

21 Rowe LA, Brillant SB, Cleveland E, *et al.* Building capacity in health facility management: guiding principles for skills transfer in Liberia. *Hum Resour Health* 2010; **8**: 5.

22 McNatt Z, Linnander E, Endeshaw A, Tatek D, Conteh D, Bradley EH. A national system for monitoring the performance of hospitals in Ethiopia. *Bull World Health Organ* 2015; **93**: 719–26.

23 Hartwig K, Pashman J, Cherlin E, *et al.* Hospital Management in the context of health sector reform: a planning

model in Ethiopia. *Int J Health Plann Manage* 2008; **23**: 203–18.
24 Kebede S, Abebe Y, Wolde M, Bekele B, Mantopoulos J, Bradley EH. Educating leaders in hospital management: a new model in Sub-Saharan Africa. *Int J Qual Health Care* 2010; **22**: 39–43.
25 Kebede S, Mantopoulos J, Ramanadhan S, *et al.* Educating leaders in hospital management: A pre-post study in Ethiopian hospitals. *Glob Public Health* 2012; **7**: 164–74.
26 Linnander E, McNatt Z, Sipsma H, *et al.* Use of a national collaborative to improve hospital quality in a low-income setting. *Int Health* 2015; **8**: 148–53.
27 Bradley E, Hartwig K a, Rowe L a, *et al.* Hospital quality improvement in Ethiopia : a partnership – mentoring model. *Int J Qual Heal Care* 2008; **20**: 392–9.
28 Tsai TC, Jha AK, Gawande AA, Huckman RS, Bloom N, Sadun R. Hospital board and management practices are strongly related to hospital performance on clinical quality metrics. *Health Aff* 2015; **34**: 1304–11.
29 Bloom N, Sadun R, Van Reenen J. Does Management Matter in Healthcare? London School of Economics Working Paper, 2013.
30 Bloom N, Propper C, Seiler S, Reenen J Van. CEP Discussion Paper No 983 May 2010 (Revised November 2014) The Impact of Competition on Management Quality : Evidence from Public Hospitals. 2013; **2010**.
31 McConnell KJ, Hoffman KA, Quanbeck A, McCarty D. Management practices in substance abuse treatment programs. *J Subst Abuse Treat* 2009; **37**: 79–89.
32 Lemos R, Scur D. Could Poor Management be Holding Back Development? Describing practices in the public and private sectors in India. 2012; : 53.
33 McConnell KJ, Lindrooth RC, Wholey DR, Maddox TM, Bloom N. Management practices and the quality of care in cardiac units. *JAMA Intern Med* 2013; **173**: 684–92.
34 Dunsch FA, Evans DK, Macis M, Giorgi G De, Loevinsohn B, Odutolu O. Management, Supervision, and Health Care: A Field Experiment. 2017.
