# Supplementary material for: Facility management associated with improved primary health care outcomes in Ghana
Source: PLoS One. 2019 Jul 2;14(7):e0218662. doi: 10.1371/journal.pone.0218662 (PMC6605853; doi:10.1371/journal.pone.0218662)
Supplement: S2 File — Complete survey used to collect data at health facilities. (PDF) [file pone.0218662.s002.pdf]

Supplementary Information 2: Facility Survey

Service Delivery Point (SDP) Questionnaire

NO	QUESTIONS AND FILTERS	CODING CATEGORIES	Relevant if:
**IDENTIFICATION**			
001	**Interviewer's name: Is this your name?**<br>[ODK will display the name associated with the phone's serial number.]<br>*Check the button next to the name if that is your name and select 'yes' here. Do not check the button if that is not your name and select 'no' here (long press to remove response next to the name if needed).*	Yes....1<br>No....0	Always
	**Enter your name below.**<br>*Please record your name*	Interviewer's Name	
002a	**Current date and time.**<br>[ODK will display on screen]<br>**Is this date and time correct?**	Yes....1<br>No....0	Always
002b	**Record the correct date and time.**	**Day** / **Month** / **Year**<br>**Hours** / **Min** / **AM/PM**	002a=0
003a	**Region**<br>*Please select the name of the Region where the facility is located.*	Ashanti....1<br>Brong-Ahafo....2<br>Central....3<br>Eastern....4<br>Greater Accra....5<br>Northern....6<br>Upper East....7<br>Upper West....8<br>Volta....9<br>Western....10	Always
003b	**District**<br>*Please select the name of the district where the facility is located.*	*ODK will populate a list of appropriate district based on the region selected for SQ E*	Always
003c	**Locality name**<br>*Please select the name of the Locality where the facility is located.*	*ODK will populate a list of appropriate localities based on the district selected for SQ F*	Always
004	**Enumeration area**	ODK will populate a list of appropriate enumeration areas based on the Locality selected	Always
005	**Facility number**<br>*Please record the number of the facility from the listing form.*	Facility number [ ]	Always

<table>
<tr><th>NO</th><th>QUESTIONS AND FILTERS</th><th>CODING CATEGORIES</th><th>Relevant if:</th></tr>
<tr><td>006</td><td>Type of facility

Please select the type of facility.</td><td>Hospital / Polyclinic......................................1
Health center 2
Health clinic 3
CHPS...4
Pharmacy...5
Chemist shop..6
Retail outlet..7
Other..8</td><td>Always</td></tr>
<tr><td>007</td><td>Managing authority

Please select the managing authority for the facility.</td><td>Government...1
NGO...2
Faith-based organization.............................3
Private...4
Other..5</td><td>Always</td></tr>
<tr><td>008</td><td>Is a competent respondent present and available to be interviewed today?</td><td>Yes...1
No ....0</td><td>Always</td></tr>
<tr><td colspan="4">INFORMED CONSENT
Find the competent respondent responsible for patient services (main administrator and family planning in-charge) who is present at the facility. Read the greeting on the next screen:</td></tr>
<tr><td colspan="4">Hello. My name is ______________________________. We are here on behalf of Kwame Nkrumah University of Science and Technology, and the Ghana Health Service to assist the government and communities in knowing more about health services. Now I will read a statement explaining the survey.

Your facility was randomly selected to participate in this study. We will be asking you questions about family planning and other reproductive health services and will ask to see patient registers. No patient names from the registers will be reviewed, recorded or shared. We will also collect information on available drugs, supplies, and equipment as well as information about provider workload, training, decision-making, and performance review. We will not need to look at or record any identifiable patient data.The information about your facility may be used by health organizations for planning service improvements or further studies of health services. The data collected from your facility will also be used by researchers for analyses. However, the name of your facility will not be provided, and any reports by researchers who use your facility data will only present information in aggregate form so that your facility cannot be identified.

We are asking for your help to ensure that the information we collect is accurate. If there are questions for which someone else is the most appropriate person to provide the information, we would appreciate your introducing us to that person.

You may refuse to answer any question or choose to stop the interview at any time. Do you have any questions about the survey?</td></tr>
<tr><td>009a</td><td>Provide a paper copy of the Consent Form to the respondent and explain it. Then, ask:
May I begin the interview now?</td><td>Yes...1
No ....0</td><td>008=1</td></tr>
<tr><td>009b</td><td>Respondent’s signature

Please ask the respondent to sign or check the box in agreement of their participation.</td><td>Gather signature:
Check box: ☐</td><td>009a=1</td></tr>
<tr><td>010</td><td>Interviewer’s name: [Interviewer name from Household Questionnaire]

Mark your name as a witness to the consent process.</td><td></td><td>009a=1</td></tr>
<tr><td>011</td><td>Name of the facility

Please record the name of the facility.</td><td></td><td>009a=1</td></tr>
</table>

NO	QUESTIONS AND FILTERS	CODING CATEGORIES	Relevant if:
012	**What is your position in this facility?**<br>*Select the highest managerial qualification of the respondent.*	Owner ....1<br>In-charge / manager ....2<br>Staff 3	009a=1
**Section 1 – Information about services**<br>*Now I would like to ask about the services provided at this facility*			
101	**What year did this facility first begin offering health services / products?**<br>*Enter Jan 2020 for do not know.*	Month [ ]<br>Year [ ]	009a=1
102	**How many days each week is the facility routinely open?**<br>*Enter a number between 0 and 7. Enter 0 for less than 1 day per week. Enter -88 for do not know, -99 for no response*	Number of days [ ]	009a=1
103	**Is there a healthcare worker present at the facility at all times or officially on call for the facility at all times (24 hours a day) for emergencies?**	Yes, 24-hr staff .... 1<br>No, no 24-hr staff.... 0<br>No response .... -99	006 ≠ 5,6,7
104	**Now I have some questions about staffing for this facility.**<br>**For the following questions, please tell me how many staff with this qualification are currently assigned to this facility.**<br>**Finally, tell me the total number present at any time today.**<br>**We want to know the highest technical qualification that any staff may hold regardless of the person's actual assignment or specialist studies.**<br>*Enter -88 for do not know and -99 for no response. 0 is a possible answer.*	(Actual # / Present today)<br>Doctor [ ] [ ]<br>Nurse/midwife [ ] [ ]<br>Medical assistant [ ] [ ]<br>Ambulance staff [ ] [ ]<br>Pharmacist [ ] [ ]<br>Medical counter assistant/ Dispensary Technician [ ] [ ]<br>Other medical staff [ ] [ ]	009a=1
105a	**Do you have an estimate of the size of the catchment population that this facility serves--that is, the target, or total population living in the area served by this facility?**	No catchment area .... 1<br>Yes, knows size of catchment area.... 2<br>Doesn't know size of catchment area .... 3<br>No response .... -99	006 ≠ 5,6,7
105b	**What is the size of the catchment population?**<br>*Record the number of people living in the area served by this facility.*	Number of people [ ]	105a=2
106	**How many beds does the facility have?**<br>*0 is a possible answer. Enter -88 for do not know, -99 for no response.*	Number of beds [ ]	006 ≠ 5,6,7
107	**When was the last time an owner / supervisor from outside this facility came here to visit?**	Never external supervision.... 0<br>Within the past 6 months.... 1<br>More than 6 months ago .... 2<br>Don't know.... -88<br>No response .... -99	009a=1

NO	QUESTIONS AND FILTERS	CODING CATEGORIES	Relevant if:
108a	**Does this facility have electricity at this time?**<br>*Select for running electricity only.*	Yes .... 1<br>No .... 0<br>No response .... -99	009a=1
108b	**At any point today, has the electricity been out for two or more hours?**	Yes .... 1<br>No .... 0<br>Don't know .... -88<br>No response .... -99	009a=1
109a	**Does this facility have running water at this time?**<br>*Select for running water only.*	Yes .... 1<br>No .... 0<br>No response .... -99	009a=1
109b	**At any point today, has running water been unavailable for two or more hours?**	Yes .... 1<br>No .... 0<br>Don't know .... -88<br>No response .... -99	009a=1
110	**How many hand-washing facilities are available on site for staff to use?**<br>*Enter -88 for do not know, -99 for no response.*	Number of facilities [ ]	006 ≠ 5,6,7
111	**May I see a nearby handwashing facility that is used by staff?**<br>*Handwashing facility must be accessible to most health workers in the facility.*<br>*At the handwashing facility, OBSERVE:*<br>*(Select all that apply.)*	Soap is present .... 1/0<br>Stored water is present .... 1/0<br>Running water is present.... 1/0<br>Handwashing area is near a sanitation facility.... 1/0<br>None of the above .... -77<br>Did not see the facility .... -99	110≠0
**Section 2 – Family Planning Service Availability**<br>*Now I would like to ask about family planning services provided at this facility.*<br>*If there is another provider who would be better able to answer my questions on family planning services in this facility, I would appreciate if you could refer me to the appropriate person.*			
201	**Do you usually offer family planning services / products?**	Yes .... 1<br>No .... 0<br>No response .... -99	009a=1
202	**What year did this facility first begin offering family planning services / products?**<br>*The respondent reported that the facility opened in [YEAR AND MONTH FROM SQ101]*<br>*Enter Jan 2020 for do not know.*	Month [ ]<br>Year [ ]	201=1
203	**How many days in a week are family planning services / products offered / sold here?**<br>**The facility is open [DAYS FROM SQ102] per week.**<br>*Enter a number between 0 and 7. Enter 0 for less than 1 day per week. Enter -88 for do not know, -99 for no response.*	Number of days [ ]	201=1
204	**Are family planning services / products offered here today?**	Yes .... 1<br>No .... 0<br>No response .... -99	201=1
205	**Does this facility provide family planning supervision, support, or supplies to community health volunteers?**	Yes .... 1<br>No .... 0<br>No response .... -99	006 = 1-4,8

NO	QUESTIONS AND FILTERS	CODING CATEGORIES	Relevant if:
206	**How many community health volunteers are supported by this facility to provide family planning services?**<br>*Record only CHVs who receive supervision, support, or supplies for family planning.*<br>*If CHVs were recorded as employees in Q103, please do not include them here as well.*<br>*Enter -88 for do not know, -99 for no response.*	Number of CHVs [ ]	205=1
207	**Do the community health volunteers provide any of the following contraceptives:**	Condoms .... 1/0<br>Pills .... 1/0<br>Injectables .... 1/0<br>None of the above .... -77<br>No response .... -99	205=1
208	**How many times in the last 6 months has a mobile outreach team visited your facility to deliver supplementary/ additional family planning services?**<br>*Enter -88 for do not know, -99 for no response. 0 is a possible answer.*	Number of times [ ]	201 = 1
209	**Which of the following family planning services do you offer to unmarried adolescents?**<br>*Read all options and select all that apply.*	Counsel for contraceptive methods........1/0<br>Provide contraceptive methods........1/0<br>Prescribe / refer contraceptive methods ...1/0<br>None of the above .... -77<br>No response .... -99	201=1

SECTOION 3 – CLIENT FEEDBACK

301	**Do you collect information about clients' opinion in any of the following ways?**<br>*Read each option out loud and select all methods that apply.*	Suggestion box ....1/0<br>Client survey form ....1/0<br>Structured interviews with clients........1/0<br>Official meeting with community leaders ...1/0<br>Informal discussion with client/ community 1/0<br>Other ....1/0<br>None of the above........-77<br>Don't know........-88<br>No response........-99	009a =1
302a	**Is there a procedure for reviewing or reporting on clients' opinions?**	Yes ....1<br>No........0	301≠ -77
302b	**Ask to see a report or form on which data are compiled or discussion is reported.**	Report seen........1<br>Report not seen........2	302= 1
303	**In the past 6 months have any changes been made in the program as a result of client opinion?**<br>*If yes, indicate if the change(s) are related to any of the listed topics.*	No........0<br>Yes, change in services or times offered or way services are provided........1<br>Yes, change for client comfort ....2<br>Other ....3<br>Don't know........-88<br>No response........-99	301 ≠ -77

SECTION 4 – PROVISION OF FAMILY PLANNING METHODS

NO	QUESTIONS AND FILTERS	CODING CATEGORIES	Relevant if:
401a	**For which of the following methods do providers at this facility counsel women about the characteristics of the method, its benefits, and its side effects?**<br><br>*Read all options out loud.*	Female sterilization .... 1/0<br>Male sterilization.................................... 1/0<br>Implant.. 1/0<br>IUD .... 1/0<br>Injectables – 3 months (Depo-Provera) . 1/0<br>Injectables – 1months (Norigynon) .... 1/0<br>Pill... 1/0<br>Emergency Contraception...................... 1/0<br>Male Condom .... 1/0<br>Female Condom .... 1/0<br>Diaphragm .... 1/0<br>Foam/Jelly .... 1/0<br>Std. Days / Cycle beads......................... 1/0<br>LAM .... 1/0<br>Rhythm method .... 1/0<br>Withdrawal.. 1/0<br>Other modern .... 1/0<br>No response .... -99	201=1
401b	**Which of the following methods are provided to clients at this facility?**<br><br>*Read all options out loud.*	Female sterilization .... 1/0<br>Male sterilization.................................... 1/0<br>Implant.. 1/0<br>IUD .... 1/0<br>Injectables – 3 months (Depo-Provera) . 1/0<br>Injectables – 1months (Norigynon) .... 1/0<br>Pill... 1/0<br>Emergency Contraception...................... 1/0<br>Male Condom .... 1/0<br>Female Condom .... 1/0<br>Diaphragm .... 1/0<br>Foam/Jelly .... 1/0<br>Std. Days / Cycle beads......................... 1/0<br>Other modern .... 1/0<br>No response .... -99	201=1
401c	**Are clients charged for obtaining any of the following methods at this facility?**<br><br>*Read all options out loud.*<br><br>*[ODK will only display methods selected in Q401b]*	Female sterilization .... 1/0<br>Male sterilization.................................... 1/0<br>Implant.. 1/0<br>IUD .... 1/0<br>Injectables – 3 months (Depo-Provera) . 1/0<br>Injectables – 1months (Norigynon) .... 1/0<br>Pill... 1/0<br>Emergency Contraception...................... 1/0<br>Male Condom .... 1/0<br>Female Condom .... 1/0<br>Diaphragm .... 1/0<br>Foam/Jelly .... 1/0<br>Std. Days / Cycle beads......................... 1/0<br>Other modern .... 1/0<br>No charge for any method...................... -77<br>No response .... -99	201=1

NO	QUESTIONS AND FILTERS	CODING CATEGORIES	Relevant if:
401d	**For which of the following methods do you provide a referral or prescription for the client to obtain the method elsewhere?**<br><br>*Read all options out loud.*<br><br>*[ODK will only display methods that were not selected in Q401b*	Female sterilization .... 1/0<br>Male sterilization.................................... 1/0<br>Implant.. 1/0<br>IUD .... 1/0<br>Injectables – 3 months (Depo-Provera) . 1/0<br>Injectables – 1months (Norigynon) .... 1/0<br>Pill.. 1/0<br>Emergency Contraception...................... 1/0<br>Male Condom .... 1/0<br>Female Condom..................................... 1/0<br>Diaphragm .... 1/0<br>Foam/Jelly .... 1/0<br>Std. Days / Cycle beads......................... 1/0<br>Other modern .... 1/0<br>No response .... -99	201=1
402	**How much do you charge for one unit of each method that you provide?**<br><br>*Enter all prices in Ghana Cedis.*<br><br>*Enter -88 for do not know, -99 for no response.*<br><br>[ODK will only display the methods for which the facility charges from Q401c.]	Amount per Unit<br>Female Sterilization (full cost of the procedure) ______<br>Male Sterilization (full cost of the procedure) ______<br>Implant (full cost of the implant and insertion) ______<br>IUD (full cost of the IUD and insertion) ______<br>One shot of the 3-month injectable (Depo-Provera) ______<br>One shot of the 1-month injectable (Norigynon) ______<br>One month supply of pills ______<br>A single dose of emergency contraception ______<br>One Male Condom ______<br>One Female Condom ______<br>Diaphragm ______<br>Foam/Jelly ______<br>Std. Days/Cycle beads ______<br>Other modern ______	401c ≠ -77
403	**Do family planning clients need to pay any fees in order to be seen by a provider in this facility even if they do not obtain a method of contraception?**<br><br>**These may be consultation or registration fees charged to everyone who is seen in this facility or may be specific to family planning clients.**	Yes .... 1<br>No 0	201=1

NO	QUESTIONS AND FILTERS	CODING CATEGORIES	Relevant if:
404	**Are the official fees posted so that the client can easily see them?**<br>*If yes, posted fees must be observed.*	Yes, all fees are posted ..... 1<br>Some, not all, fees posted ..... 2<br>No posted fees ..... 0<br>No response ..... -99	403=1
405	**On days when you offer family planning services, does this facility have trained personnel able to insert implants?**	Yes ..... 1<br>No ..... 0<br>No response ..... -99	006 ≠ 5,6,7 and 401b: implant= 1
406	**On days when you offer family planning services, does this facility have trained personnel able to remove implants?**	Yes ..... 1<br>No ..... 0<br>No response ..... -99	006 ≠ 5,6,7 and 401b: implant= 1
407	**On days when you offer family planning services, does this facility have trained personnel able to insert IUDs?**	Yes ..... 1<br>No ..... 0<br>No response ..... -99	006 ≠ 5,6,7 and 401b: IUD=1
408	**On days when you offer family planning services, does this facility have trained personnel able to remove IUDs?**	Yes ..... 1<br>No ..... 0<br>No response ..... -99	006 ≠ 5,6,7 and 401b: IUD=1
409	**Does this facility have the following supplies needed to insert and/or remove implants:**<br>*Read out all supplies and select all that apply. Supplies do not need to be observed, but must be available on the day of the interview.*	Clean Gloves ..... 1/0<br>Antiseptic ..... 1/0<br>Sterile Gauze Pad or Cotton Wool ..... 1/0<br>Local anaesthetic ..... 1/0<br>Sealed Implant Pack ..... 1/0<br>Surgical Blade ..... 1/0<br>None of the above ..... -77<br>No response ..... -99	401b: implant= 1
410	**Does this facility have the following supplies needed to insert and/or remove IUDs:**<br>*Read out all supplies and select all that apply. Supplies do not need to be observed, but must be available on the day of the interview.*	Sponge-holding forceps ..... 1/0<br>Speculums (large and medium) ..... 1/0<br>Tenaculum ..... 1/0<br>Clamp ..... 1/0<br>None of the above ..... -77<br>No response ..... -99	401b: IUD=1

NO	QUESTIONS AND FILTERS	CODING CATEGORIES	Relevant if:
411a	**From family planning register, record:**<br><br>(1) The total number of family planning visits (new and continuing) in the last completed month, for each method.<br>(2) The number of new clients who received family planning services in the last completed month, for each method.<br>*Past completed month. Enter -88 for no not know, enter -99 for no response.*	Method / Total # visits / # new clients:<br>Female Sterilization ___ ___<br>Male Sterilization ___ ___<br>Implants ___ ___<br>IUD ___ ___<br>Injectables-3 month (Depo-Provera) ___ ___<br>Injectables-1 month (Norigynon) ___ ___<br>Pill ___ ___<br>Emergency contraception ___ ___<br>Male Condom ___ ___<br>Female Condom ___ ___<br>Diaphragm ___ ___<br>Foam/Jelly ___ ___<br>Std. Days/Cycle beads ___ ___<br>Other modern ___ ___	006 = 1-4, 8
411b	**From family planning record book, record:**<br>The total number of family planning products sold in the last completed month, for each method.<br>*The total number of family planning products sold in the last completed month, for each method.*<br>*Enter -88 for do not know, enter -99 for no response.*	Method / # of units sold or provided:<br>Female Sterilization ___<br>Male Sterilization ___<br>Implants ___<br>IUD ___<br>Injectables-3 month (Depo-Provera) ___<br>Injectables-1 month (Norigynon) ___<br>Pill ___<br>Emergency contraception ___<br>Male Condom ___<br>Female Condom ___<br>Diaphragm ___<br>Foam/Jelly ___<br>Std. Days/Cycle beads ___<br>Other modern ___	006 = 5,6,7
412	**In the past 6 months, have there been any meetings where service statistics (or inventory) for family planning are discussed with staff?**	Yes....1<br>No....0<br>No response....-99	201=1

NO	QUESTIONS AND FILTERS	CODING CATEGORIES	Relevant if:
413	**May I see any wall charts, graphs, written reports, minutes or other written materials produced with service data from the past 12 months?**<br>*Select all relevant types of documentation observed. Posters or other IEC materials that do not contain any service data should not be counted.*	Observed wall chart / graph .... 1/0<br>Observed written report / minutes .... 1/0<br>Observed other means of reviewing service data .... 1/0<br>Other .... 1/0<br>Nothing observed .... -77	009a=1 AND 412=1
414a	**May I see the room where examinations for family planning are conducted?**	Yes .... 1<br>No .... 0<br>No response .... -99	201=1
414b	**For each of the following items, check to see whether item is either in room where examinations are conducted or in an adjacent room.**	See table below	414a=1
415	**OBSERVE: Assess condition of family planning service area**<br>*Must answer all of the above or none of the above.*	Floors: swept, no obvious dirt or waste.. 1/0<br>Surfaces: wiped clean, no obvious dirt or waste .... 1/0<br>Area is tidy and uncluttered .... 1/0<br>Walls: reasonably clean .... 1/0<br>Doors: no or minor damage .... 1/0<br>Walls: no or minor damage .... 1/0<br>Roof: no or minor damage 1/0	414a=1
416a	**You mentioned that you typically provide the [METHOD] at this facility, can you show it to me?**<br>**If no, probe: Is the [METHOD] out of stock today?**<br>[238a-c will repeat for each of the methods that are provided at the facility according to SQ 31, except Female and Male Sterilization]	In-stock and observed .... 1<br>In-stock but not observed .... 2<br>Out of stock .... 3<br>No Response .... -99	201=1

414b items:

Item	Observed	Reported but unseen	Not available
Running water (piped)	1	2	-77
Other running water (bucket with tap or pour pitcher)	1	2	-77
Water in bucket or basin (water reused)	1	2	-77
Hand-washing soap	1	2	-77
Single-use hand drying towels	1	2	-77
Waste receptacle with lid and plastic liner	1	2	-77
Sharps container	1	2	-77
Disposable latex gloves	1	2	-77
Disinfectant	1	2	-77
Disposable needles and syringes	1	2	-77
Auditory privacy	1	2	-77
Visual privacy	1	2	-77
Examination table	1	2	-77
Client educational materials on FP	1	2	

NO	QUESTIONS AND FILTERS	CODING CATEGORIES			Relevant if:
416b	**How many days has the [METHOD] been out of stock?**<br>[52a-c will repeat for each of the methods that are provided at the facility according to SQ 31, except Female and Male Sterilization]<br>Enter 1 if only for today.<br>Enter -88 for Do not know.<br>Enter -99 for No response.	Number of days ☐			417a=3
416c	**Has the [METHOD] been out of stock at any time in the last 3 months?**<br>[52a-c will repeat for each of the methods that are provided at the facility, except Female and Male Sterilization]	Yes .... 1<br>No .... 0<br>Don't know .... -88<br>No response .... -99			417a=1 or 2
417a	**May I see the room where contraceptive supplies are stored?**<br>If you are already in the room, select "Yes"	Yes .... 1<br>No 0			201=1
417b	**Observe the place where contraceptive supplies are stored and report on the following condition:**<br>Are all the methods off the floor?<br>Are all the methods protected from water?<br>Are all the methods protected from the sun?<br>Is the room clean of evidence of rodents (bats, rats) or pests (roaches)?		Yes<br>1<br>1<br>1<br>1	No<br>0<br>0<br>0<br>0	417a=1
**SECTION 5: FAMILY PLANNING SERVICE INTEGRATION**					
501	**Which of the following services are provided at this facility:**<br>*Read all options and select all that apply.*	Antenatal .... 1/0<br>Delivery .... 1/0<br>Postnatal .... 1/0<br>Post-abortion .... 1/0<br>None of the above .... -77<br>No response .... -99			009a=1
502	**Which of the following is discussed with the mother after delivery or during the first postnatal visit:**<br>*Read all options and select all that apply.*<br>*If your respondent is not involved in delivery or postnatal care, ask if they can refer you to someone at the facility who provides these services.*	Return to fertility .... 1/0<br>Healthy timing and spacing of pregnancies .... 1/0<br>Immediate and exclusive breastfeeding 1/0<br>Family planning methods available to use while breastfeeding .... 1/0<br>Lactational Amenorrhea Method and transition to other methods .... 1/0<br>Long-acting method options .... 1/0<br>None of the above .... -77<br>No response .... -99			501: Del=1<br>OR<br>Postnat= 1
503	**Is the woman offered a method of family planning during the postnatal visit?**	Yes .... 1<br>No .... 0<br>No response .... -99			501: postnat = 1

NO	QUESTIONS AND FILTERS	CODING CATEGORIES	Relevant if:
504	**During post-abortion visits, which of the following is discussed with the client:**<br>*Read all options and select all that apply. If your respondent is not involved in post-abortion care, ask if they can refer you to someone at the facility who provides these services.*	Post-abortion mental health .... 1/0<br>Return to fertility .... 1/0<br>Healthy timing and spacing of pregnancies .... 1/0<br>Long-acting method options.................. 1/0<br>FP methods for birth spacing .... 1/0<br>None of the above .... -77<br>No response .... -99	501: Post-abortion= 1
505	**Is the woman offered a method of family planning during the post-abortion visit?**	Yes .... 1<br>No 0	501: Post-abortion= 1
506	**Does this facility offer any service related to diagnosis, treatment, or supportive services for HIV?**	Yes .... 1<br>No .... 0<br>No response .... -99	009a=1
507	**Does this facility offer any service related to diagnosis, treatment, or supportive services for STIs other than HIV?**	Yes .... 1<br>No .... 0<br>No response .... -99	009a=1
508a	**When a client comes in for HIV services, are they offered condoms by the HIV service provider?**<br>*If your respondent is not involved in HIV service provision, ask if they can refer you to someone at the facility who provides these services.*	Yes .... 1<br>No .... 0<br>Don't know... -88<br>No response .... -99	506=1 AND 006a≠ 5-7
508b	**Does the HIV service provider offer them any other method of contraception besides condoms?**	Yes .... 1<br>No .... 0<br>Don't know... -88<br>No response .... -99	506=1 AND 006a≠ 5-7
508c	**Are they given information on where they can obtain contraception elsewhere?**	Yes .... 1<br>No .... 0<br>Don't know... -88<br>No response .... -99	508b=0 AND 006a≠ 5-7
508d	**Are they referred within the facility, outside the facility, or both?**	Within facility only..................................... 1<br>Outside facility only .... 2<br>Both .... 3<br>Don't know... -88<br>No response .... -99	508c=1 AND 006a≠ 5-7

PRIMARY HEALTH CARE MODULE

NOTE: If you are at a hospital, you should seek out the Medical Director/Medical Superintendent of the facility. If he/she is not available, you may also speak to the Director of Nursing/Matron. If both individuals are not present, you will need to reschedule a time to return to the facility to complete this module.

If you are in a Health Center, the respondent should be the Nurse in-charge, Midwife in-charge, or Physician in-charge.

At a CHPS facility, the respondent should be the Community Health Nurse.

At any private facility, you should first speak to the owner to confirm that they are the correct respondent or if someone else is. Acceptable respondents include: the owner or managing partner, the administrator, and/or the highest-ranking doctor in the facility.

PHC 002	**What is your position in this facility?**<br>*Select the highest managerial qualification of the respondent.*	Owner (private facility only) .... 1<br>Managing partner (private facility only) .... 1<br>Administrator .... 1<br>Medical Director... 1<br>Medical Superintendent................................ 1<br>Deputy Director of Nursing/Matron............... 1<br>Nurse/Midwife In-Charge.............................. 1<br>Physician Assistant In-Charge .... 1<br>Community Health Nurse<br>Staff 3	009a=1
PHC 003	**Does this facility have a separate general outpatient department?**	Yes.. 1<br>No 0	009a=1

SECTION 1: POPULATION HEALTH MANAGEMENT (PHC 101 – 107)

NOTE: If you are at a hospital, the appropriate respondent is the Medical Director/Medical Superintendent of the facility. If he/she is not available, you may also speak to the Director of Nursing/Matron. If both individuals are not present, you will need to reschedule a time to return to the facility to complete this section. This person may refer you to other individuals to answer specific questions, which is acceptable.

If you are in a Health Center, the respondent should be the Nurse in-charge, Midwife in-charge, or Physician in-charge.

At a CHPS facility, the respondent should be the Community Health Nurse.

At any private facility, acceptable respondents include the owner or managing partner and/or the highest-ranking doctor in the facility.

PHC 101a	**Is your facility accountable for the health outcomes of a group of people, even if they never attend services at your health facility?**<br><br>*IF NEEDED: By health outcome, we mean things like maternal mortality, vaccination coverage, or hypertension control.*	Yes ....<br>..<br>..1<br>No ....<br>..0<br>Do not know... -88<br>No response .... -99	PHC 001a=1
PHC 101b	**How many people?**<br>*Record numeric value*	________________	PHC 101a=1
PHC 102	**How do you know who this population is?**<br>*Do not read the answer choices to the respondent, but mark all choices that the respondent mentions.*<br><br>*Examples:*<br>*Geographic enrollment: assigned by the MOH, example CHPS zone*<br>*Voluntary enrollment: patients sign up to receive care from this facility or simply choose to seek care at the facility*	Based on geographic assignment.................1<br>By voluntary enrollment................................2<br>Other..4<br>Do not know... -88	PHC 101a=1
PHC 103	**Are you required to measure coverage of key population indicators, such as immunization coverage?**<br>*IF NEEDED: other examples of population indicators include maternal mortality, ANC coverage, and hypertension control.*	Yes ....1<br>No ....0<br>Do not know... -88<br>No response .... -99	PHC 001a=1

PHC 104	**Does this facility regularly receive reports tracking the most common health conditions and outcomes in the community?**<br>*If needed for clarification, you may explain that "regular reports mean that there is a system in place to receive these reports at regular intervals (weekly, monthly, etc.) regardless of any extreme outcomes such as infant mortality or new disease outbreak." Reports may come from the district, regional, national levels or from the community and/or lower-level health facilities.*	Yes ....1<br>No ....0<br>Do not know.... -88<br>No response .... -99	PHC 001a=1
PHC 105	**Do you have a mechanism to collect and report new disease outbreaks?**<br>*If the respondent is unsure, you may prompt with "For example, is there a mechanism in this facility to track new incidence of diseases such as cholera, yellow fever, or meningitis?"*	Yes ....1<br>No ....0<br>Do not know.... -88<br>No response .... -99	PHC 001a=1
PHC 106	**What methods are used to gather information on health outcomes and new disease outbreaks tracked by the facility?**<br>*Do not read options. Mark all that apply.*	Community Health Workers routinely capture and report information on priority areas ....1<br>Disease-based registries at the facility ....2<br>Staff Review of active patients with priority conditions who visit the facility ....3<br>Government surveys of the community ....4<br>Routine DHIMS ....5<br>Other surveys of the community ....6<br>Other....7<br>No response .... -99	PHC 104=1 or PHC 105=1
PHC 107	**Are the results of the health conditions and outcomes collected and shared with facility staff through any means?**<br>*Do not read options. Mark all that apply.*	Displayed in the facility (chalkboard, poster, noticeboard)....1/0<br>Staff meetings ....1/0<br>On individual basis as requested ....1/0<br>Other....1/0<br>Not collected/shared....1/0<br>Do not know.... -88<br>No response .... -99	PHC 104=1 or PHC 105=1
PHC 108	**Does this facility provide supervision, support, or supplies to community health workers?**	Yes ....1<br>No ....0<br>No response .... -99	PHC 001a=1
PHC 109	**How many community health workers are supported by this facility to provide any health services?**<br><br>*Enter -88 for do not know*<br>*Enter -99 for no response*	Number of CHWs [ ]	PHC108=1

PHC 110	**Which of the following types of community health outreach activities do the Community Health Workers that are supported by this facility conduct?**<br>*Read all options out loud.*<br>*Select all that apply.*	ANC counseling..1/0<br>Provide immunizations/vaccinations ....1/0<br>Family planning counseling........................1/0<br>Family planning provision (ex: condoms)...1/0<br>Postnatal care ....1/0<br>Water, Sanitation, and Hygiene counseling (WASH)...1/0<br>Mental health counseling ....1/0<br>Non-communicable disease treatment ......1/0<br>Disease surveillance ....1/0<br>Active case finding ....1/0<br>Directly Observed Therapy (DOT) for TB ..1/0<br>C-IMCI/iCCM (Community Integrated Management of Childhood Illness/Integrated Community Case Management).............1/0<br>Health education ....1/0<br>Community mobilization ....1/0<br>Enrollment in the facility ....1/0<br>Outreach for loss to follow-up ....1/0<br>Other...1/0<br>None of the above .... -77<br>Do not know... -88<br>No response .... -99	PHC108=1
PHC 111	**Who is in charge of supervising CHW activities?**<br>*Do not read options out loud. Mark all that apply.*	Community Health Officer/Nurse ....1<br>Public Health Nurse......................................2<br>Midwife ....3<br>Health Assistant (community) ....4<br>Physician Assistant/Medical Assistant ....5<br>No Response... -99	PHC108=1
PHC 112	**How frequently do CHWs receive supervision?**<br><br>*Do not read options out loud. Record the response in the unit the respondent provides, ie "Once per X [days/weeks/months/years]."*	○ X Days<br>○ X Weeks<br>○ X Months<br>○ X Years<br>No response	PHC 108a=1

SECTION 2: PROVIDER WORKLOAD (PHC 201 – 202)

NOTE: If you are at a hospital, the appropriate respondent is the Medical Director/Medical Superintendent of the facility. If he/she is not available, you may also speak to the Director of Nursing/Matron. If both individuals are not present, you will need to reschedule a time to return to the facility to complete this section. This person may refer you to other individuals to answer specific questions, which is acceptable.

If you are in a Health Center, the respondent should be the Nurse in-charge, Midwife in-charge, or Physician in-charge.

At a CHPS facility, the respondent should be the Community Health Nurse.

At any private facility, acceptable respondents include the owner or managing partner and/or the highest-ranking doctor in the facility.

PHC 201a	**In your opinion, what is the average length of time, in minutes, spent by a provider in a typical consultation with a patient in this [facility/outpatient department]?**<br>*"Provider" means any clinical staff who hold consultations with patients.*<br>*Enter your answer in minutes. If the respondent does not know the exact amount of time, ask them to make their best guess.*<br>*Enter -88 for Do not know*<br>*Enter -99 for No response*	Minutes	PHC 001a=1
PHC 202a	**May I see your patient register in order to note down the total number of patients seen in this [facility/outpatient department] yesterday?**	Yes ....1<br>No ....0<br>No record exists .... -88<br>No response .... -99	PHC 001a=1
PHC 202b	**Count the number of visits in the register(s) from the day before the interview.**<br><br>*You should record all visits that occurred in the health facility (or, at hospitals, in the OPD) on the LAST DAY BEFORE YOUR INTERVIEW that the facility/OPD was open. If there are multiple registers, you should count all patient visits from all registers and record the total.*<br><br>*Enter -88 for Do not know*<br>*Enter -99 for No response*	Patient visits ☐	PHC 202a=1
PHC 202c	**What is your best estimate of the number of patients seen in this [facility/outpatient department] yesterday?**<br>*You should ask the respondent to make their best estimate of how many patients were seen in the facility/OPD the LAST DAY BEFORE YOUR VISIT that the facility/OPD was open.*<br><br>*Enter -88 for Do not know*<br>*Enter -99 for No response*	Patient visits ☐	PHC 202a≠ 1 or PHC 202b=-88, -99

SECTION 3: SUPERVISION AND PROFESSIONAL DEVELOPMENT (PHC 301 – 308)

NOTE: If you are at a hospital, the appropriate respondent is the Medical Director/Medical Superintendent of the facility. If he/she is not available, you may also speak to the Director of Nursing/Matron. If both individuals are not present, you will need to reschedule a time to return to the facility to complete this section. This person may refer you to other individuals to answer specific questions, which is acceptable.

If you are in a Health Center, the respondent should be the Nurse in-charge, Midwife in-charge, or Physician in-charge.

At a CHPS facility, the respondent should be the Community Health Nurse.

At any private facility, acceptable respondents include the owner or managing partner and/or the highest-ranking doctor in the facility.

PHC 301	**In the past 12 months, have supervisors at your facility held individual meetings with staff to review their performance?**	Yes ....1<br>No ....0<br>Do not know .... -88<br>No response .... -99	PHC 001a=1

PHC 302	**Which categories of staff receive performance reviews?**<br>*Read the options out loud and select all that apply*	Nurses ....1/0<br>Doctors ....1/0<br>Disease control officers ....1/0<br>Health education officers ....1/0<br>Nutrition officers ....1/0<br>Laboratory staff ....1/0<br>Pharmaceutical staff ....1/0<br>Administrative staff……………………….1/0<br>Janitorial staff ....1/0<br>Managers...1/0<br>Community health workers affiliated with the facility ....1/0<br>Midwives ....1/0<br>Other...1/0	PHC 301=1
PHC 303	**Do you have a set of established criteria your facility uses to evaluate staff performance?**	Yes ....1<br>No ....0<br>Do not know... -88<br>No response .... -99	PHC 001a=1
PHC 304	**Which of the following criteria are used?**<br>*Read options aloud. Select all that apply.*	Direct staff supervision while looking after a patient ....1/0<br>Health worker absenteeism record ....1/0<br>Timeliness ....1/0<br>Health worker average caseload................1/0<br>Patient satisfaction ....1/0<br>Patient outcomes.......................................1/0<br>Knowledge assessment ....1/0<br>Staff attitude ....1/0<br>Staff investment in the facility....................1/0<br>Proper adherence to protocols……………..1/0<br>Other...1/0	PHC 303=1
PHC 305	**What is the main method of supervision in place in your facility?**<br>*Do not read options out loud. Select the one option that best matches the response.*	Formal supervision process with regular pre-arranged supervision meetings ....1<br>Supervision is only available if requested by staff ....2<br>Supervision is supportive and continuous.....3<br>Supervision consists of negative feedback when performance is poor ....4<br>Other...6<br>None, no method of supervision ....5<br>No response .... -99	PHC 001a=1
PHC 306	**Are staff in the facility offered trainings to improve their skills?**	Yes ....<br>..<br>..1<br>No ....0<br>No response .... -99	PHC 001a=1
PHC 307	**Which of the following methods does your facility use to decide who should have access to in-service training?**<br><br>*Read each option out loud and ask the respondent to tell you whether this method is used in the facility or not. Tick all that apply.*	Opportunity to go for in-service training is decided by supervisor/manager ....1 / 0<br>Training opportunities are offered based on a formal review of training needs of each employee ....1 / 0<br>Training opportunities are offered based on job performance review (focusing on skills required for the job) ....1 / 0<br>No specific process ....1 / 0<br>Other...1 / 0	PHC 406=1

<table>
<tr><td>PHC 308</td><td>How many clinical staff have left (quit, retired, moved, transferred, etc) within the last 6 months?
If the respondent is unsure of the exact number, you should ask them to make their best estimate.
Enter -88 for Do not know
Enter -99 for No response</td><td>Number who have left: ______</td><td>PHC 001a=1</td></tr>
<tr><td colspan="4">Section 4: Facility Management (PHC 401 – 414)
NOTE: If you are at a hospital, the appropriate respondent is the Medical Director/Medical Superintendent of the facility. If he/she is not available, you may also speak to the Director of Nursing/Matron. If both individuals are not present, you will need to reschedule a time to return to the facility to complete this section. This person may refer you to other individuals to answer specific questions, which is acceptable.

If you are in a Health Center, the respondent should be the Nurse in-charge, Midwife in-charge, or Physician in-charge.

At a CHPS facility, the respondent should be the Community Health Nurse.

At any private facility, acceptable respondents include the owner or managing partner and/or the highest-ranking doctor in the facility.</td></tr>
<tr><td>PHC 401</td><td>Have you ever received any formal training in the management of a health facility?</td><td>Yes 1
No 0
No response .... -99</td><td>PHC 001a=1</td></tr>
<tr><td>PHC 402</td><td>What type of formal training in management have you received?
Do not read out loud. Select all that apply
Prompt: “Are there any other types of health facility management training you have attended or obtained?”</td><td>A short course (a week or less)...............1 / 0
A short course (Between a week and a month) ....1 / 0
A short course (Between one month and six months) ....1 / 0
One or more courses as part of my medical degree ....1 / 0
One or more courses as a part of my undergraduate/diploma training ....1 / 0
Technical degree in management...........1 / 0
Graduate degree in management ....1 / 0
Post graduate degree in management ....1 / 0
Other...1 / 0
No response .... -99</td><td>PHC 501=1</td></tr>
<tr><td>PHC 403a</td><td>How many total hours did you work [yesterday] at this facility?
Clarify that we want the total number of hours the respondent worked the last day that they worked at this facility. Let the respondent know that an estimate is ok if they are not sure.
Enter -88 for Do not know
Enter -99 for No response</td><td>Total hours: ______</td><td>PHC 001a=1</td></tr>
</table>

PHC 403b	**During this day, how much time did you devote to each of these activities?**<br>*Record in hours*<br><br>**a. Overseeing patient flow** *(e.g., patient admissions, triage, transfers, and discharges)* [ ]<br><br>**b. Supervising medical staff** *(e.g., meeting with staff, providing feedback, checking absenteeism.)* [ ]<br><br>**c. Managing operational budgets** *(e.g., tracking revenue, submitting claims, paying bills)* [ ]<br><br>**d. Verifying/Ensuring availability of drugs and equipment** *(e.g., taking inventory, placing orders, etc.)* [ ]<br><br>**e. Treating patients yourself** *(e.g., providing consultations)* [ ]<br><br>**f. Managing relationships with staff, community, facility committee, donors, and government**<br>**g. other** [ ]	PHC 001a=1

In this part of the questionnaire, I would like to know what you would do if a certain hypothetical situation were to arise at this facility. I will read you a series of scenarios. For each scenario, I will read 4 possible responses that you might have. Please let me know the response that most closely matches what you would do in this specific situation, even if it is not exactly what you would want to do. You can only select one response for each scenario. There are no correct or incorrect answers—we just want to know how you would approach each situation.

PHC 404	**Scenario 1: A health worker often does not come to work on Mondays because he/she travels to another village during the weekend. Which of the following actions would you take?**<br>*Read the following response choices and ask the respondent to select the one that MOST matches what they would do in this hypothetical scenario. Remind them there is no correct answer, and that we are simply interested in learning how they would approach the situation.*	You approve his/her absence and get a replacement for those days ....1<br><br>You tell him/her that this is not acceptable and if this behavior persists you will request his/her transfer (internal or external) ....2<br><br>You attempt to facilitate transportation and reduce the time that he/she is absent from the facility...3<br><br>You approve his/her absence and ask him/her to work extra hours to compensate. ....4<br><br>Other...5	PHC 001a=1
PHC 405	**Scenario 2: A recent assessment of the facility found that a health worker does not follow the adequate clinical guidelines for patient treatment.**<br>*Read the following response choices and ask the respondent to select the one that MOST matches what they would do in this hypothetical scenario. Remind them there is no correct answer, and that we are simply interested in learning how they would approach the situation.*	You request to transfer him/her (internal or external)..1<br><br>You demand him/her to improve his performance. If it does not improve, you request a transfer. (internal or external)........2<br><br>You allow him/her freedom to set his own goals for improvement and do not push him. 3<br><br>You send him/her to get additional training or arrange more supervision and then monitor his/her progress..4<br><br>Other...5	PHC 001a=1

PHC 406	**Scenario 3: You notice that in the last few months the facility has frequently run out of antibiotics for children. Which of the following actions would you take?**<br>*Read the following response choices and ask the respondent to select the one that MOST matches what they would do in this hypothetical scenario. Remind them there is no correct answer, and that we are simply interested in learning how they would approach the situation.*	You purchase the medicines in town with your own money or internally generated funds then have them available for purchase by the patients who need them. ....1<br><br>You instruct clinicians in your staff to ask patients to buy the medicines themselves in a pharmacy in town. ....2<br><br>You request the government headquarters for more stocks. ....3<br><br>You inform the government of the stock out and ask the community for support in collecting funds to purchase extra stocks of these medicines. ....4<br><br>Other...5	PHC 001a=1
Now I would like to ask you some questions about who has the most authority to make specific decisions for this facility. The options for each question are Ghana Health Services, the Director of the facility/In-Charge, a Health facility/Management Committee, Doctors or staff of the facility, the community, or other. For each decision, please tell me who has the most say. There are no correct or incorrect answers to these questions—we only want to understand your experiences.			
PHC 407a	**According to you, which of these groups has the most say in deciding when to order more drugs in the facility?**<br>*Read the answer choices out loud and as the respondent to pick who has the MOST say in this decision.*	Ghana Health Service ....1<br>Director of the facility/In-charge ....2<br>Health facility/Hospital Management Committee ....3<br>Doctors/facility staff ....4<br>Community ....5<br>Other...6<br>No response .... -99	PHC 001a=1
PHC 407b	**How much say do you have in this decision?**<br>*Read the answer options out loud and ask the respondent to pick the most appropriate response.*	None ....1<br>A little...2<br>A lot ....3<br>No response .... -99	PHC 001a=1
PHC 408a	**According to you, which of these groups has the most say in deciding on recruitment of health workers for the facility?**<br>*Read the answer choices out loud and as the respondent to pick who has the MOST say in this decision.*	Ghana Health Service ....1<br>Director of the facility/In-charge ....2<br>Health facility/Hospital Management Committee ....3<br>Doctors/facility staff ....4<br>Community ....5<br>Other...6<br>No response .... -99	PHC 001a=1
PHC 408b	**How much say do you have in this decision?**<br>*Read the answer options out loud and ask the respondent to pick the most appropriate response.*	None ....1<br>A little...2<br>A lot ....3<br>No response .... -99	PHC 001a=1
PHC 409a	**According to you, which of these groups has the most say in deciding which health workers get promoted?**<br>*Read the answer choices out loud and as the respondent to pick who has the MOST say in this decision.*	Ghana Health Service ....1<br>Director of the facility/In-charge ....2<br>Health facility/Hospital Management Committee ....3<br>Doctors/facility staff ....4<br>Community ....5<br>Other...6<br>No response .... -99	PHC 001a=1

PHC 409b	**How much say do you have in this decision?**<br>*Read the answer options out loud and ask the respondent to pick the most appropriate response.*	None ....1<br>A little ....2<br>A lot ....3<br>No response .... -99	PHC 001a=1
PHC 410a	**According to you, which of these groups has the most say in taking disciplinary action against health workers?**<br>*Read the answer choices out loud and as the respondent to pick who has the MOST say in this decision.*	Ghana Health Service ....1<br>Director of the facility/In-charge ....2<br>Health facility/Hospital Management Committee ....3<br>Doctors/facility staff ....4<br>Community ....5<br>Other....6<br>No response .... -99	PHC 001a=1
PHC 410b	**How much say do you have in this decision?**<br>*Read the answer options out loud and ask the respondent to pick the most appropriate response.*	None ....1<br>A little ....2<br>A lot ....3<br>No response .... -99	PHC 001a=1
PHC 411a	**According to you, which of these groups has the most say in deciding to paint a wall or fix the refrigerator in the facility?**<br>*Read the answer choices out loud and as the respondent to pick who has the MOST say in this decision.*	Ghana Health Service ....1<br>Director of the facility/In-charge ....2<br>Health facility/Hospital Management Committee ....3<br>Doctors/facility staff ....4<br>Community ....5<br>Other....6<br>No response .... -99	PHC 001a=1
PHC 411b	**How much say do you have in this decision?**<br>*Read the answer options out loud and ask the respondent to pick the most appropriate response.*	None ....1<br>A little ....2<br>A lot ....3<br>No response .... -99	PHC 001a=1
PHC 412a	**According to you, which of these groups has the most say in approving health worker absence?**<br>*Read the answer choices out loud and as the respondent to pick who has the MOST say in this decision.*	Ghana Health Service ....1<br>Director of the facility/In-charge ....2<br>Health facility/Hospital Management Committee ....3<br>Doctors/facility staff ....4<br>Community ....5<br>Other....6<br>No response .... -99	PHC 001a=1
PHC 412b	**How much say do you have in this decision?**<br>*Read the answer options out loud and ask the respondent to pick the most appropriate response.*	None ....1<br>A little ....2<br>A lot ....3<br>No response .... -99	PHC 001a=1
PHC 413a	**According to you, which of these groups has the most say in setting service delivery priorities for the health facility?**<br>*Read the answer choices out loud and as the respondent to pick who has the MOST say in this decision.*	Ghana Health Service ....1<br>Director of the facility/In-charge ....2<br>Health facility/Hospital Management Committee ....3<br>Doctors/facility staff ....4<br>Community ....5<br>Other....6<br>No response .... -99	PHC 001a=1
PHC 413b	**How much say do you have in this decision?**<br>*Read the answer options out loud and ask the respondent to pick the most appropriate response.*	None ....1<br>A little ....2<br>A lot ....3<br>No response .... -99	PHC 001a=1

PHC 414a	**According to you, which of these groups has the most say in how to spend internally generated funds at the facility?** *Read the answer choices out loud and as the respondent to pick who has the MOST say in this decision.*	Ghana Health Service ....1<br>Director of the facility/In-charge ....2<br>Health facility/Hospital Management Committee ....3<br>Doctors/facility staff ....4<br>Community ....5<br>Other....6<br>No response .... -99	PHC 001a=1
PHC 414b	**How much say do you have in this decision?** *Read the answer options out loud and ask the respondent to pick the most appropriate response.*	None ....1<br>A little....2<br>A lot ....3<br>No response .... -99	PHC 001a=1

SECTION 5: MONITORING AND QUALITY IMPROVEMENT (PHC 501 – 504)

NOTE: If you are at a hospital, the appropriate respondent is the Medical Director/Medical Superintendent of the facility. If he/she is not available, you may also speak to the Director of Nursing/Matron. If both individuals are not present, you will need to reschedule a time to return to the facility to complete this section. This person may refer you to other individuals to answer specific questions, which is acceptable.

If you are in a Health Center, the respondent should be the Nurse in-charge, Midwife in-charge, or Physician in-charge.

At a CHPS facility, the respondent should be the Community Health Nurse.

At any private facility, acceptable respondents include the owner or managing partner and/or the highest-ranking doctor in the facility.

PHC 501	**Does this facility conduct any quality improvement activities?**	Yes ....1<br>No ....0<br>No response .... -99	PHC 001a=1
PHC 502	**Who is responsible for conducting quality improvement activities in this facility?** *Read the available answer choices and ask the respondent to select the most appropriate option.*	No specific group....1<br>Facility leader ....2<br>A specific group of staff (can include the facility leader or not) ....3<br>Responsibility shared across all staff and made explicit through dedicated meeting time ....4<br>External staff (from NGO, government, etc.) 5<br>Other....6<br>No response .... -99	PHC 501=1
PHC 503	**In the past 12 months, have there been any meetings where routinely collected service statistics or clinical audit data are discussed with staff?**	Yes ....1<br>No ....0<br>No response .... -99	PHC 001a=1

PHC 504	**How much do you agree or disagree with the following statement: The use of data to monitor and improve service delivery is highly valued in this facility.**<br>*Read the available answer choices and ask the respondent to select the most appropriate option.*	Strongly Agree....1<br>Agree....2<br>Disagree....4<br>Strongly Disagree....5<br>No response.... -99	PHC 001a=1

SECTION 6: COMMUNITY ENGAGEMENT (PHC 601 – 606)

NOTE: If you are at a hospital, the appropriate respondent is the Medical Director/Medical Superintendent of the facility. If he/she is not available, you may also speak to the Director of Nursing/Matron. If both individuals are not present, you will need to reschedule a time to return to the facility to complete this section. This person may refer you to other individuals to answer specific questions, which is acceptable.

If you are in a Health Center, the respondent should be the Nurse in-charge, Midwife in-charge, or Physician in-charge.

At a CHPS facility, the respondent should be the Community Health Nurse.

At any private facility, acceptable respondents include the owner or managing partner and/or the highest-ranking doctor in the facility.

PHC 601	**Does this facility have a community advisory board or community management committee that meets regularly?**	Yes....1<br>No....0<br>Do not know.... -88<br>No response.... -99	PHC 001a=1
PHC 602	**Has the facility taken any follow-up action on the basis of discussions had during the last meeting?**	Yes....1<br>No....0<br>Do not know.... -88<br>No response.... -99	PHC 701=1
PHC 603	**Is there a community member who regularly attends staff meetings?**	Yes....1<br>No....0<br>No staff meetings are held.... -77<br>No response.... -99	PHC 001a=1
PHC 604	**In the past 12 months, has this facility shared information on its performance with the community it serves?**	Yes....1<br>No....0<br>No response.... -99	PHC 001a=1
PHC 605	**How do you share this type of information?**<br>*Read the answer choices out loud and select all that apply*	Chalk boards/Noticeboards....1 /0<br>Posters....1 /0<br>Newsletters....1 /0<br>Community events at the facility/in the community....1 /0<br>Community Health Workers....1 /0<br>Community Advisory Board or Management Committee....1 / 0<br>Do not know....1 /0<br>Other....1 /0<br>No response.... -99	PHC 604=1

PHC 606	**How much do you think that patients' opinions about their experiences at your facility drive change or improvement efforts?**<br>*Read the answer choices out loud and ask the respondent to select the option that best matches their opinion.*	Nearly always ....1<br>Very often ....2<br>Sometimes..3<br>Rarely ....4<br>Never ....5<br>No response .... -99	PHC 001a=1
**SECTION 7: SUPPLIES, EQUIPMENT, AND ESSENTIAL DRUGS (PHC 701 – 710)**<br>*NOTE: If you are at a facility with an outpatient department, you should ask the respondent to refer you to the physician or nurse in-charge of the outpatient department for this section.*<br><br>*If the facility does not have a separate outpatient department, you may continue with the current respondent.*			
PHC 701	**Now I have some questions about staffing in this [facility/outpatient department].**<br>**For the following questions, please tell me how many staff with this qualification are working at any time today in the [facility/outpatient department] .**<br>**We want to know the highest technical qualification that any staff may hold regardless of the person's actual assignment or specialist studies.**<br>*Enter -88 for do not know and -99 for no response. 0 is a possible answer.*	Present today<br>Doctor ____<br>Nurse/midwife ____<br>Medical assistant ____<br>Pharmacist ____<br>Other medical staff ____	PHC003 =1
PHC 702a	**Does this [facility/outpatient department] have a functional (working today) ambulance or other vehicle that is available for emergency transportation?**	Yes ....1<br>No ....0<br>Do not know.. -88<br>No response .... -99	PHC 001a=1
PHC 702b	**Is fuel available today?**	Yes ....1<br>No ....0<br>Do not know.. -88<br>No response .... -99	PHC 702a=1
Now I would like to ask you about the materials and resources available in this [facility/outpatient department]. For each of the items I am interested in knowing if it is available in the [facility/outpatient department] today and if it is functioning properly. I will ask to see each of the items mentioned.			
PHC 703a	**Does this [facility/outpatient department] have an adult weighing scale available today?**<br><br>**Probe: If so, can you show it to me?**	Observed ....1<br>Reported, but unseen...................................2<br>Not Available ....3<br>No response .... -99	PHC 001a=1
PHC 703b	**Is it functioning properly?**	Yes, functioning..1<br>No, not functioning ....0<br>Appears to be significantly damaged 2	PHC 703a=1, 2
PHC 704a	**Does this [facility/outpatient department] have a child or infant weighing scale available today?**<br><br>**Probe: If so, can you show it to me?**	Observed ....1<br>Reported, but unseen...................................2<br>Not Available ....3<br>No response .... -99	PHC 001a=1
PHC 704b	**Is it functioning properly?**	Yes, functioning..1<br>No, not functioning ....0<br>Appears to be significantly damaged 2	PHC 704a=1, 2

PHC 705a	**Does this [facility/outpatient department] have a sphyg (blood pressure cuff) available today?**<br><br>**Probe: If so, can you show it to me?**	Observed ....1<br>Reported, but unseen ....2<br>Not Available ....3<br>No response .... -99	PHC 001a=1
PHC 705b	**Is it functioning properly?**	Yes, functioning ....1<br>No, not functioning ....0<br>Appears to be significantly damaged 2	PHC 705a=1, 2
PHC 706a	**Does this [facility/outpatient department] have a thermometer available today?**<br><br>**Probe: If so, can you show it to me?**	Observed ....1<br>Reported, but unseen ....2<br>Not Available ....3<br>No response .... -99	PHC 001a=1
PHC 706b	**Is it functioning properly?**	Yes, functioning ....1<br>No, not functioning ....0<br>Appears to be significantly damaged 2	PHC 706a=1, 2
PHC 707a	**Does this [facility/outpatient department] have a Stethoscope available today?**<br><br>**Probe: If so, can you show it to me?**	Observed ....1<br>Reported, but unseen ....2<br>Not Available ....3<br>No response .... -99	PHC 001a=1
PHC 707b	**Is it functioning properly?**	Yes, functioning ....1<br>No, not functioning ....0<br>Appears to be significantly damaged 2	PHC 707a=1, 2
PHC 708	**Does this [facility/outpatient department] have any of the following sterilization equipment available and functioning?**<br>*Clarify that this equipment can be located anywhere in the [facility/outpatient department]. Read options out loud. Check all that apply. You do not need to observe the equipment.*	Autoclave ....1 / 0<br>Electric boiler or steamer ....1 / 0<br>Electric dry heat sterilizer ....1 / 0<br>Incinerator ....1 / 0<br>None of the above .... -77<br>No response .... -99	PHC 001a=1
PHC 709	**Does the [facility/outpatient department] have non-electric sterilization equipment?**<br>*Clarify that this equipment can be located anywhere in the [facility/outpatient department]*<br>**Probe: If so, can you show it to me?**	Observed ....1<br>Reported, but unseen ....2<br>Not Available ....3<br>No response .... -99	PHC 001a=1
PHC 710	**Are the following non-expired drugs available in this [facility/outpatient department] today?**<br>*Ask about each medication separately. You do not need to observe.*	Oxytocin ....1 / 0<br>Misoprostol (cap/tab) ....1 / 0<br>Sodium chloride (saline solution)/(injection solution) .... 1 / 0<br>Azithromycin (cap/tab or oral liquid) ....1 / 0<br>Calcium gluconate (injectable) ....1 / 0<br>Magnesium sulfate ....1 / 0<br>Ampicillin powder (for injection) ....1 / 0<br>Betamethasone or Dexamethansone (injectable) ....1 / 0<br>Gentamicin (injectable) ....1 / 0<br>Nifedipine (cap/tab) ....1 / 0<br>Metronidazole (injectable) ....1 / 0<br>Iron supplements (cap/tab) ....1 / 0<br>Folic acid supplements (cap/tab) ....1 / 0<br>Amoxicillin (syrup/suspension) ....1 / 0<br>Oral Rehydration Salts (ORS sachets) ....1 / 0<br>Zinc (tablets) ....1 / 0<br>Ceftriaxone (injectable) ....1 / 0<br>Artemisinin combination therapy (ACT) ....1 / 0<br>Artusunate (rectal or injectable) ....1 / 0<br>Benzylpenicillin (powder for injection) ....1 / 0<br>Vitamin A (capsules) ....1 / 0<br>None of the above .... -77<br>No response .... -99	PHC 001a=1

**SECTION 8: INFORMATION SYSTEM USE (PHC 801 – 802)**<br>*NOTE: If you are at a facility with an outpatient department and have been speaking to the in-charge in this unit, you should now ask to speak to the highest-ranking administrator, Medical Director/Medical Superintendent, or the Director of Nursing/Matron. If these individuals are not present, you will need to reschedule a time to return to the facility to complete this section.*<br><br>*If you are in a facility that does not have an outpatient department, you may continue speaking to the current respondent OR seek out the highest-ranking administrator at the facility.*<br><br>*At any private facility, acceptable respondents include the owner or managing partner, the highest-ranking administrator, and/or the highest-ranking doctor in the facility.*			
PHC 801	**Does each patient at your health facility have one single unique and accessible health record that follows her/him over time and departments?**<br><br>*NOTE: Select "Yes with 1 or 2 exceptions" if the facility maintains one individual patient record for all types of services except one or two, such as ANC.*	Yes ....1<br>Yes, with one or two exceptions..........2<br>No ....0<br>No response .... -99	PHC 001a=1
PHC 802a	**May I see one of these patient health records?**<br><br>**PROBE: Are there any other formats used for these health records? May I see them?**<br><br>*Do not read out loud. Observe the record(s) and select all formats that apply.*	Paper-based record maintained at the facility ....1/0<br>Paper-based record maintained and brought to the facility by the patient..........1/0<br>Electronic record ....1/0<br>Other..........1/0<br>No record available to view ....1/0<br>No response .... -99	PHC 801=1,2
PHC 802b	**Can you describe the format of the health record to me?**<br>*Do not read out loud. Select all that apply.*	Paper-based record maintained at the facility ....1/0<br>Paper-based record maintained and brought to the facility by the patient..........1/0<br>Electronic record ....1/0<br>Other..........1/0<br>Do not know.......... -88<br>No response .... -99	"No record available to view" Selected in 302a or 802a=-99
**SECTION 9: FINANCING (PHC 901 – 913)**<br>*NOTE: You should continue speaking to the highest-ranking administrator at the facility for this question. If you are in a Health Center, the respondent may be the Nurse in-charge, Midwife in-charge, or Physician in-charge. At a CHPS facility, the respondent may be the Community Health Nurse. At any private facility, acceptable respondents include the owner or managing partner and/or the highest-ranking doctor in the facility.*<br><br>*The respondent may refer you to someone in the financing/accounting department for these questions, which is acceptable.*			
Now I would like to ask you a few questions about the financing, revenue, and expenditures of this facility.			
PHC 901	**Are any user fees/charges displayed at the facility?**<br><br>**Probe: May I see where they are displayed?**<br>*Record only if observed.*	Observed ....1<br>Reported, but unseen..........2<br>Not Available ....3<br>No response .... -99	PHC 001a=1

PHC 902	**In your facility, which of the following groups is exempt from paying user fees out of pocket?**<br>*Read options out loud and select all that apply.*	Patients with chronic diseases ....1 / 0<br>Elderly patients ....1 / 0<br>Very poor people ....1 / 0<br>Facility staff...1 / 0<br>Relatives of staff ....1 / 0<br>Members of health management board ...1 / 0<br>Local politicians ....1 / 0<br>Children under five years ....1 / 0<br>Women seeking maternal or family planning services..1 / 0<br>People covered by NHIS ....1 / 0<br>Other...1 / 0<br>No one .... -77<br>No response .... -99	PHC 001a=1
PHC 903	**Does your facility have one comprehensive annual budget for running costs? By running costs, I mean all of the costs of operating this facility, including paying staff, building maintenance, and purchasing of supplies, equipment, medicines, and utilities.**	Yes .... 1<br>No ....0<br>Do not know... -88<br>No response .... -99	PHC 001a=1
PHC 904	**Does your facility maintain books to track revenue and expenditures?**<br><br>**Probe: May I see the books?**	Observed ....1<br>Reported, but unseen...................................2<br>Not Available ....3<br>Do not know... -88<br>No response .... -99	PHC 001a=1
PHC 905	**What was the total amount of internally generated funds for the facility in the last fiscal year?**<br>*By "last fiscal year" we mean the last fiscal year which has been fully completed, not the current fiscal year.*<br>*If respondent does not know, ask if there is somewhere they can look it up. If not, ask them to make their best estimate.*<br>*Record in [GHC]*<br>*Enter -88 for Do not know*<br>*Enter -99 for No response*	Total: ______	PHC 001a=1
PHC 906	**Were the internally generated funds collected in this facility during the last fiscal year used to pay for any of the following?**<br>*Ask about each type of expenditure separately and select "yes" or "no."*	Medicines ....1/0<br>Medical Supplies (syringes, gauze, gloves, etc.) ....1/0<br>Medical equipment (thermometer, stethoscope, sphygmomanometer, etc.) .1/0<br>Cleaning supplies (detergents, mops, etc).1/0<br>Salaries of any health workers ....1/0<br>Salaries of support staff (cleaner, gardener, guard, etc.)1 / 0<br>Allowances ....1/0<br>Utilities (electricity, water, gas, telephone).1/0<br>Building construction/ maintenance ....1/0<br>Vehicles ....1/0<br>Other...1/0<br>No internally generated funds used .... -77<br>Do not know... -88<br>No response .... -99	PHC 001a=1
PHC 907	**In the last 12 months, has there been an external financial audit of your facility?**	Yes .... 1<br>No ....0<br>Do not know... -88<br>No response .... -99	PHC 001a=1

PHC 908	**Is this facility NHIS approved?**<br><br>*If needed: NHIS approved means that the facility is eligible to receive payments from the NHIS for services it provides.*	Yes ....1<br>No ....0<br>Do not know.. -88<br>No response .... -99	PHC 001a=1
PHC 909	**How much time does it typically take for this facility to receive reimbursements for NHIS claims?**<br>*Enter using the units given by the respondent.*	X Days...1<br>X Weeks...2<br>X Months ....3<br>X Years..4<br><br>Enter # Days: ____<br>Enter # Weeks: ____<br>Enter # Months: ____<br>Enter # Years: ____	PHC908=1

LOCATION AND QUESTIONNAIRE RESULT			
094	**Ask permission to take a photo of the entrance of the facility.**<br><br>**Did you get consent to take the photo?**	Yes .... 1<br>No 0	009a=1
**Thank the respondent for her / his time.**<br>*The respondent is finished, but there are still more questions for you to complete outside the facility.*			
095	**Ensure that no people are in the photo**	TAKE PICTURE<br><br>CHOOSE IMAGE	094=1
096	**Location**<br><br>**Take a GPS point outside near the entrance to the facility. Record location when the accuracy is smaller than 6m.**	RECORD LOCATION	Always
097	**How many times have you visited this service delivery point for this interview?**	1st time .... 1<br>2nd time .... 2<br>3rd time 3	Always
098	**In what language was this interview conducted?**	English .... 1<br>Akan .... 2<br>Ga .... 3<br>Ewe .... 4<br>Nzema .... 5<br>Dagbani .... 6<br>Other 96	009a=1
099	**Record the result of the Service Delivery Point Questionnaire.**	Completed .... 1<br>Not at facility .... 2<br>Postponed .... 3<br>Refused .... 4<br>Partly completed .... 5<br>Other 6	Always
