# Supplementary material for: Facility management associated with improved primary health care outcomes in Ghana
Source: PLoS One. 2019 Jul 2;14(7):e0218662. doi: 10.1371/journal.pone.0218662 (PMC6605853; doi:10.1371/journal.pone.0218662)
Supplement: S3 File — Complete survey used to collect data from individual women in their households. (PDF) [file pone.0218662.s003.pdf]

Supplementary Information 3: Household Survey—Female Questionnaire

Female Questionnaire

NO	QUESTIONS AND FILTERS	CODING CATEGORIES	Relevant If:
**IDENTIFICATION**			
001a	**Are you in the correct household?**<br>**This is the picture of the front of the home taken during the Household Questionnaire.**<br>[ODK will display the photo attached to the linked Household Questionnaire]	Yes.. 1<br>No 0	Always
001b	**RETURN TO INTERVIEW THE CORRECT HOUSEHOLD.**		001a=0
002	**Your name:**<br>[Interviewer name from Household Questionnaire]<br>**Is this your name?**	Yes.. 1<br>No 0	Always
	**Enter your name below.**<br>*Please record your name*	Interviewer's Name	002=0
003	**Current date and time.** [ODK will display on screen]<br>**Is this date and time correct?**	Yes.. 1<br>No 0	Always
	**Record the correct date and time.**	**Day** / **Month** / **Year**: ___ / ___ / ___<br>**Hours** / **Min** / **AM/PM**: ___ / ___ / ___	003=0
004a	**The following information is from the Household Questionnaire. Please review to make sure you are interviewing the correct respondent.**<br>[ODK will display the *Region, District, Locality,* Enumeration Area, Structure Number, and Household Number entered into the Household Questionnaire linked to this Female Questionnaire.]<br>**Is the above information correct?**	Yes.. 1<br>No 0	Always
	**GO TO THE RIGHT HOUSEHOLD OR UPDATE THE HOUSEHOLD ROSTER IF NEEDED.**		004a = 0
005	**CHECK: You should be attempting to interview [Respondent's Name]. Is that correct?**<br>*If misspelled, select "yes" here and update the name in question "011."*<br>*If this is the wrong person, you have two options:*	Yes.. 1<br>No 0	Always

	(1) exit and ignore changes to this form. Open the correct form.<br>*Or*<br>*(2) find and interview the person whose name appears above.*		
006	**Is the respondent present and available to be interviewed today?**	Yes.......... 1<br>No 0	Always
007	**How well acquainted are you with the respondent?**	Very well acquainted .... 1<br>Well acquainted .... 2<br>Not well acquainted .... 3<br>Not acquainted.......... 4	006 = 1
008	**Has the respondent previouslyparticipated in PMA2020 surveys?**	Yes.......... 1<br>No .... 0<br>Don't know ....-88<br>No response ....-99	006 = 1

**INFORMED CONSENT**<br>*Find the woman between the ages of 15-49 associated with this Female Questionnaire. The interview must have auditory privacy. Read the following greeting:*			
Hello. My name is ____________________ and I am working for Kwame Nkrumah University of Science and Technology, and the Ghana Health Service. We are conducting a local survey that asks women about various reproductive health issues. *We shall also ask you questions about your experiences seeking healthcare for yourself or your children. This information will help us understand how people use the health services available, for what purpose, and whether they are of high or low quality.* We would very much appreciate your participation in this survey. This information will help us inform the government to better plan health services. The survey usually takes between 20 and minutes to complete. Whatever information you provide will be kept strictly confidential and will not be shown to anyone other than members of our survey team.<br><br>Participation in this survey is voluntary, and if we should come to any question you don't want to answer, just let me know and I will go on to the next question; or you can stop the interview at any time. However, we hope that you will participate in this survey since your views are important.<br>At this time, do you want to ask me anything about the survey?			
009a	Provide a paper copy of the Consent Form to the respondent and explain it. Then, ask:<br>**May I begin the interview now?**	Yes ....1<br>No..........0	006 =1
009b	**Respondent's signature**<br>*Please ask the respondent to sign or check the box in agreement of their participation.*	GATHER SIGNATURE:<br><br>Checkbox: ☐	009a = 1
010	**Interviewer's name:** [Interviewer name from Household Questionnaire]<br>*Mark your name as a witness to the consent process.*		009a = 1
011	**Respondent's first name**<br>[ODK will display the Respondent's name from linked Household Roster]<br>*You may correct the spelling here if it is not correct, but you must be interviewing the person whose name appears below.*		009a = 1

**Section 1 – Respondent's Background, Marital Status, and household characteristics**<br>*Now I would like to ask about your background and socioeconomic conditions.*			
101	**In what month and year were you born?**<br>**The age in the household roster is [AGE].**	Month ____<br>Year ____	009a = 1
102	**How old were you at your last birthday?**<br>*Must be more than 14. Must agree with FQ101.*	Age ____	009a = 1
103	**What is the highest level of school you attended?**	Never Attended ....0<br>Primary ....1<br>Middle / JSS ....2<br>Secondary / SSS ....3<br>Higher ....4<br>No response ....-99	009a = 1
LCL 101	**Do you have any health insurance or are you a member of a mutual health organization?**	Yes ....1<br>No ....0<br>No response ....-99	009a = 1
LCL 102	**What type of health insurance do you have?**<br><br>*Record all that are mentioned*	National/District Health Insurance (NHIS) ....1<br>Health insurance through employer ....2<br>Mutual health organization/ Community-based health insurance ....3<br>Other privately purchased commercial health insurance ....4<br>Other ....5<br>No Response ....-99	LCL101 =1
LCL 103	**Do you hold a valid National Health Insurance Scheme (NHIS) card?**<br><br>*If answer is "Yes", request to see the card.*	Yes, card seen ....1<br>Yes, card not seen/lost ....2<br>No ....3<br>No Response ....-99	LCL102 =1
104	**Are you currently married or living together with a man as if married?**<br>*Probe: If no, ask whether the respondent is divorced, separated, or widowed.*	Yes, currently married ....1<br>Yes, living with a man ....2<br>Not currently in union:<br>Divorced / separated ....3<br>Not currently in union: Widow ....4<br>No, never in union ....5<br>No response ....-99	009a = 1
105	**Have you been married or lived with a man only once or more than once?**	Only once ....1<br>More than once ....2<br>No response ....-99	009a = 1 AND 104 ≠ 5
106a	**In what month and year did you start living with your FIRST husband / partner?**<br>*Enter Jan 2020 for no response.*	Month ____<br>Year ____	105=2
106b	**CHECK: Based on the response you entered in 106a, the respondent was possibly 15 years old or**	Yes ....1<br>No 0	

	**younger at the time of her first marriage. Did you enter 106a correctly?**		106a age at marriage ≤15
106c	**RETURN TO 106A TO CORRECT BEFORE CONTINUING**		106b = 0
107a	**Now I would like to ask about when you started living with your CURRENT or MOST RECENT husband / partner. In what month and year was that?**<br>*Enter Jan 2020 for no response.*	Month [ ]<br>Year [ ]	105 = 1 or 2
107b	[If ≤15 years old at marriage date ODK will display:] **CHECK: Based on the response you entered in107a, the respondent was possibly 15 years old or younger at the time of her first marriage. Did you enter 107a correctly?**	Yes ....1<br>No 0	107a age at marriage ≤15
107c	**RETURN TO 107A TO CORRECT BEFORE CONTINUING**		107b = 0
108	**Does your husband / partner have other wives or does he live with other women as if married?**	Yes ....1<br>No ....0<br>Don't know ....-88<br>No response ....-99	104 = 1 or 2
109	**Is your husband / partner living with you now or is he staying elsewhere?**	Living with respondent ....1<br>Staying elsewhere ....2<br>No response ....-99	104 = 1 or 2
**Section 2 – Reproduction, Pregnancy & Fertility Preferences**			
200	**Now I would like to ask about all the births you have had during your life. Have you ever given birth?**	Yes ....1<br>No ....0<br>No response ....-99	009a=1
201a	**Do you have any sons or daughters to whom you have given birth who are now living with you?**	Yes ....1<br>No ....0<br>No response ....-99	009a =1 AND 200=1
201b	**How many sons live with you?**<br>*Zero is a possible response. Enter -99 for No response.*	Number [ ]	201a = 1
201c	**How many daughters live with you?**<br>*Zero is a possible response. Enter -99 for No response.*	Number [ ]	201a = 1
202a	**Do you have any sons or daughters to whom you have given birth who are alive but do not live with you?**	Yes ....1<br>No ....0<br>No response ....-99	009a=1 AND 200 = 1
202b	**How many sons are alive but do not live with you?**<br>*Zero is a possible response. Enter -99 for No response.*	Number [ ]	202a = 1

202c	**How many daughters are alive but do not live with you?**<br>*Zero is a possible response. Enter -99 for No response.*	Number ☐	202a = 1
203a	**Have you ever given birth to a boy or girl who was born alive but later died?**<br>*IF NO, PROBE: Any baby who cried, who made any movement, sound, or effort to breathe, or who showed any other signs of life even if for a very short time?*	Yes ....1<br>No...0<br>No response.................................-99	009a = 1
203b	**How many boys have died?**<br>*Zero is a possible response. Enter -99 for No response.*	Number ☐	203a = 1
203c	**And how many girls have died?**<br>*Zero is a possible response.*<br>*Enter -99 for No response.*	Number ☐	203a = 1
CALC _CEB	CALCULATE: NUMBER OF CHILDREN EVER BORN ODK will automatically calculate the total number of children ever born from FQ201-203: (201b+201c+202b+202c+203b+203c)	Number ☐	
204	**Just to make sure that I have this right, you have had in TOTAL [NUMBER OF CHILDREN EVER BORN] births during your life. Is that correct?**	Yes ....1<br>No...0	CALC _CEB > 0
205	**When was your FIRST birth?**<br>*Please record the date of the first live birth. Date should be found by calculating forward or backward from memorable events if needed. Enter Jan 2020 for no response.*	Month ☐<br>Year ☐	CALC _CEB >1
206	**When was your MOST RECENT birth?**<br>*Please record the date of the MOST RECENT live birth. The date should be found by calculating backwards from memorable events if needed.*<br>*Enter Jan 2020 for no response.*	Month ☐<br>Year ☐	CALC _CEB >0
207	**When did you give birth before the most recent one?**<br>*Please record the date of the birth before the last. The date should be found by calculating backwards from memorable events if needed.*<br>*Enter Jan 2020 for no response.*	Month ☐<br>Year ☐	CALC _CEB >2
208a	**Is your last baby / child still alive?**	Yes ....1<br>No...0<br>Don't know ....-88<br>No response.................................-99	(201a = 1 OR

			202a = 1) AND 203a = 1
208b	**When did your last baby / child die?**<br>*Please record the date of the child's death. The date should be found by calculating backwards from memorable events if needed. Enter Jan 2020 for no response.*	Month [ ]<br>Year [ ]	208a = 0 or (201a=0 and 202a=0 and 203a=1)
209	**When did your last menstrual period start?**<br>*If you select days, weeks, months or years, you will enter a number for x on the next screen.*<br>*Enter 0 days for today, not 0 weeks/months/years.*	[ ] days ago<br>[ ] weeks ago<br>[ ] months ago<br>[ ] years ago<br>Menopausal / Hysterectomy ....5<br>Before last birth ....6<br>Never menstruated ....7<br>No response.................................-99	009a = 1
210a	**Are you pregnant now?**	Yes ....1<br>No..0<br>Unsure...2<br>No response.................................-99	009a = 1 AND 209 ≠ 5
210b	**How many months pregnant are you? The most recent birth was: [Date of most recent birth]**<br>*Please record the number of completed months. Enter -88 for do not know, -99 for no response.*	Number of months [ ]	210a = 1
211a	**Now I have some questions about the future. Would you like to have a/another child or would you prefer not to have any / any more children?**	Have a/another child ....1<br>No more/prefer no children ....2<br>Says she can't get pregnant ....3<br>Undecided / Don't know ....-88<br>No response.................................-99	009a =1 AND 210a ≠ 1 AND CALC_CEB ≤ 0
211b	**Now I have some questions about the future. After the child you are expecting now, would you like to have another child, or would you prefer not to have any more children?**	Have a/another child ....1<br>No more/prefer no children ....2<br>Says she can't get pregnant ....3<br>Undecided / Don't know ....-88<br>No response.................................-99	210a = 1
212a	**How long would you like to wait from now before the birth of a/another child?**<br>*If you select months or years, you will enter a number for x on the next screen.*<br>*Select "Years" if more than 36 months.*<br>*Please check that you correctly entered the value for months/years.*	Months [ ]<br>Years [ ]<br>Soon / now ....3<br>Other ....4<br>Says she can't get pregnant 5	211a = 1

		Don't know ....-88<br>No response.................................-99	
212b	**After the birth of the child you are expecting now, how long would you like to wait before the birth of another child?**<br>*If you select months or years, you will enter a number for x on the next screen.*<br>*Select "Years" if more than 36 months.*<br>*Please check that you correctly entered the value for months/years.*	Months [ ]<br>Years [ ]<br>Soon / now ....3<br>Says she can't get pregnant ....4<br>Other ....5<br>Don't know ....-88<br>No response.................................-99	211b = 1
213a	**Now I would like to ask a question about your last birth.**<br>**At the time you became pregnant, did you want to become pregnant then, did you want to wait until later, or did you not want to have any / any more children at all?**	Then ....1<br>Later ....2<br>Not at all ....3<br>No response.................................-99	CALC _CEB>0 AND 210a ≠ 1
213b	**Now I would like to ask a question about your current pregnancy.**<br>**At the time you became pregnant, did you want to become pregnant then, did you want to wait until later, or did you not want to have any / any more children at all?**	Then ....1<br>Later ....2<br>Not at all ....3<br>No response.................................-99	210a = 1
**Section 3 – Contraception**<br>*Now I would like to talk about family planning - the various ways or methods that a couple can use to delay or avoid a pregnancy.*<br>*An image will appear on the screen for some methods. If the respondent says that she has not heard of the method or if she hesitates to answer, read the probe aloud and show her the image, if available.*			
301a	**Have you ever heard of female sterilization?**<br>PROBE: Women can have an operation to avoid having any more children.<br>[NO IMAGE]	Yes ....1<br>No..0<br>No response……………………….-99	009a = 1
301b	**Have you ever heard of male sterilization?**<br>PROBE: Men can have an operation to avoid having any more children.<br>[NO IMAGE]	Yes ....1<br>No..0<br>No response……………………….-99	009a = 1
301c	**Have you ever heard of the contraceptive implant?**<br>PROBE: Women can have one or several small rods placed in their upper arm by a doctor or nurse, which can prevent pregnancy for one or more years.<br>[IMAGE OF METHOD WILL APPEAR ON SCREEN]	Yes ....1<br>No..0<br>No response……………………….-99	009a = 1
301d	**Have you ever heard of the IUD?**	Yes 1	009a = 1

	PROBE: Women can have a loop or coil placed inside them by a doctor or a nurse.<br><br>[IMAGE OF METHOD WILL APPEAR ON SCREEN]	No..0<br>No response………………………..-99	
301e	**Have you ever heard of injectables?**<br><br>PROBE: Women can have an injection by a health provider that stops them from becoming pregnant for one or more months.<br><br>[IMAGE OF METHOD WILL APPEAR ON SCREEN]	Yes ....1<br>No..0<br>No response………………………..-99	009a = 1
301f	**Have you ever heard of the (birth control) pill?**<br><br>PROBE: Women can take a pill every day to avoid becoming pregnant.<br><br>[IMAGE OF METHOD WILL APPEAR ON SCREEN]	Yes ....1<br>No..0<br>No response………………………..-99	009a = 1
301g	**Have you ever heard of emergency contraception?**<br><br>PROBE: As an emergency measure after unprotected sexual intercourse women can take special pills at any time within five days to prevent pregnancy.<br><br>[NO IMAGE]	Yes ....1<br>No..0<br>No response………………………..-99	009a = 1
301h	**Have you ever heard of condoms?**<br><br>PROBE: Men can put a rubber sheath on their penis before sexual intercourse.<br><br>[IMAGE OF METHOD WILL APPEAR ON SCREEN]	Yes ....1<br>No..0<br>No response………………………..-99	009a = 1
301i	**Have you ever heard of female condoms?**<br><br>PROBE: Women can put a sheath in their vagina before sexual intercourse.<br><br>[IMAGE OF METHOD WILL APPEAR ON SCREEN]	Yes ....1<br>No..0<br>No response………………………..-99	009a = 1
301j	**Have you ever heard of the diaphragm?**<br><br>PROBE: Women can place a thin flexible disk in their vagina before sexual intercourse.<br><br>[IMAGE OF METHOD WILL APPEAR ON SCREEN]	Yes ....1<br>No..0<br>No response………………………..-99	009a = 1
301k	**Have you ever heard of foam or jelly as a contraceptive method?**	Yes ....1<br>No..0<br>No response………………………..-99	009a = 1

	PROBE: Women can place a suppository, jelly, or cream in their vagina before sexual intercourse to prevent pregnancy.<br><br>[IMAGE OF METHOD WILL APPEAR ON SCREEN]		
301l	**Have you ever heard of the standard days method or Cycle Beads?**<br><br>PROBE: A Woman can use a string of colored beads to know the days she can get pregnant. On the days she can get pregnant, she and her partner use a condom or do not have sexual intercourse.<br><br>[IMAGE OF METHOD WILL APPEAR ON SCREEN]	Yes ....1<br>No...0<br>No response………………………-99	009a = 1
301 m	**Have you ever heard of the Lactational Amenorrhea Method or LAM?**<br><br>[NO DESCRIPTION; NO IMAGE]	Yes ....1<br>No...0<br>No response………………………..-99	009a = 1
301n	**Have you ever heard of the rhythm method?**<br><br>PROBE: Women can avoid pregnancy by not having sexual intercourse on the days of the month they think they can get pregnant.<br><br>[NO IMAGE]	Yes ....1<br>No...0<br>No response………………………..-99	009a = 1
301o	**Have you ever heard of the withdrawal method?**<br><br>PROBE: Men can be careful and pull out before climax.<br><br>[NO IMAGE]	Yes ....1<br>No...0<br>No response………………………..-99	009a = 1
301p	**Have you ever heard of any other ways or methods that women or men can use to avoid pregnancy?**	Yes ....1<br>No...0<br>No response………………………..-99	009a = 1
302a	**Are you or your partner currently doing something or using any method to delay or avoid getting pregnant?**	Yes ....1<br>No...0<br>No response………………………..-99	210a ≠ 1 AND 009a=1

302b	**Which method or methods are you using?**<br>**Probe: Anything else?**<br>*Select all methods mentioned. SCROLL TO THE BOTTOMto see all choices.*	Female sterilization .... 1/0<br>Male sterilization .... 1/0<br>Implant .... 1/0<br>IUD .... 1/0<br>Injectables-3 month....... 1/0<br>Injectables-1 month....... 1/0<br>Pill....... 1/0<br>Emergency Contraception .... 1/0<br>Male Condom....... 1/0<br>Female Condom .... 1/0<br>Diaphragm....... 1/0<br>Foam/Jelly....... 1/0<br>Std. Days/Cycle beads....... 1/0<br>LAM....... 1/0<br>N_tablet....... 1/0<br>Rhythm method....... 1/0<br>Withdrawal .... 1/0<br>Washing .... 1/0<br>Other traditional methods....... 1/0<br>No response……………………….-99	302a = 1
CALC _CM	ODK CALCULATE: CURRENT METHOD<br>THIS WILL NOT APPEAR ON THE SCREEN.<br>ODK Will identify the most effective method currently being used by the respondent by selecting the highest method in the choice list.	Female Sterilization .... 1<br>Male Sterilization....... 2<br>Implant .... 3<br>IUD .... 4<br>Injectables-3 month....... 5<br>Injectables-1 month....... 6<br>Pill....... 7<br>Emergency Contraception .... 8<br>Male Condom....... 9<br>Female Condom .... 10<br>Diaphragm....... 11<br>Foam/Jelly....... 12<br>Std. Days/Cycle beads....... 13<br>LAM....... 14<br>N-tablet....... 15<br>Rhythm method....... 30<br>Withdrawal .... 31<br>Washing .... 32<br>Other traditional methods....... 39<br>No response……………………….-99	302a=1 & 302b ≠ -99
302c	**Are you breastfeeding to delay or avoid becoming pregnant?**	Yes .... 1<br>No....... 0<br>No Response……………………… -99	LAM selected in 302b
303	**Did the provider tell you or your partner that this method was permanent?**	Yes .... 1<br>No....... 0<br>No response……………………….-99	Female or Male Sterilization selected in 302b
304	**Do you know of a place where you can obtain a method of family planning?**	Yes .... 1<br>No....... 0<br>No response……………………….-99	009a=1 AND (210a=1 OR 302a≠1)

305a	**You said that you are not currently using a contraceptive method. Do you think you will use a contraceptive method to delay or avoid getting pregnant at any time in the future?**	Yes....1<br>No....0<br>No response....-99	009a=1 AND 302a ≠ 1 AND 210a ≠ 1
305b	**Do you think you will use a contraceptive method to delay or avoid getting pregnant at any time in the future?**	Yes....1<br>No....0<br>No response....-99	210a = 1 AND 302a ≠ 1 AND 210a=1
306a	**In the last 12 months, have you ever done something or used a method to delay or avoid getting pregnant?**	Yes....1<br>No....0<br>No response....-99	009a=1 AND (210a = 1 OR 302a ≠ 1)
306b	**Which method did you use most recently?**<br>**Probe: Anything else?**<br>*Select most effective method (highest method on list). Scroll to bottom to see all choices.*	Implant....3<br>IUD....4<br>Injectables – 3 month....5<br>Injectables – 1 month....6<br>Pill....7<br>Emergency Contraception....8<br>Male Condom....9<br>Female Condom....10<br>Diaphragm....11<br>Foam/Jelly....12<br>Std. Days/Cycle beads....13<br>LAM....14<br>N_tablet....15<br>Rhythm method....30<br>Withdrawal....31<br>Washing....32<br>Other traditional methods....39<br>No response....-99	306a = 1
307	**Before you started using [MOST RECENT / CURRENT METHOD], had you discussed the decision to delay or avoid pregnancy with your husband/partner?**	Yes....1<br>No....0<br>Don't know....-88<br>No response....-99	302a = 1 OR 306a = 1
308	**Would you say that using contraception is mainly your decision, mainly your husband/partner's decision or did you both decide together?**	Mainly respondent....1<br>Mainly husband/partner....2<br>Joint Decision....3<br>Other....96<br>No response....-99	009a=1 AND (302a=1 OR 306a=1)
309a	**Since what month and year have you been using [CURRENT METHOD] without stopping?**<br>*Calculate backwards from memorable events if needed.*<br>**Most Recent Birth: [mm-yyyy]**<br>**Current Marriage: [mm-yyyy]**	Month [ ]<br>Year [ ]	302a=1

	Must be before today. Respondent must be at least 10 years old.<br><br>*Enter Jan 2020 for no response.*		
309b	**When did you stop using your [MOST RECENT METHOD]?**<br><br>*Please record the date.*<br><br>*The date should be found by calculating backwards from memorable events if needed.*<br><br>*Enter Jan 2020 for no response.*	Month ☐<br>Year ☐	306a=1
309c	**In what month and year had you started using [MOST RECENT METHOD] before stopping?**<br><br>*Calculate backwards from memorable events if needed.*<br><br>**Most Recent Birth: [mm-yyyy]**<br>**Current Marriage: [mm-yyyy]**<br><br>*Must be before today. Respondent must be at least 10 years old.*<br><br>*Enter Jan 2020 for no response.*<br><br>*Enter Jan 2020 for no response..*	Month ☐<br>Year ☐	306a=1
309d	**CHECK: Just to make sure I have this correct, you used [RECENT METHOD] continuously between [START DATE] and [END DATE] without stopping, is that correct?**	Yes ....1<br>No 0	306a=1
	**GO BACK TO THE PREVIOUS SCREEN AND PROBE TO DETERMINE THE PERIOD OF MOST RECENT CONTINUOUS USE.**<br><br>*Suggested probes:*<br>- *When was the last time you used [METHOD]?*<br>- *How long had you been using [METHOD] without stopping*		310d = 0
310	**Why did you stop using your [MOST RECENT METHOD]?**	Infrequent sex / husband away........1<br>Became pregnant while using..........2<br>Wanted to become pregnant ....3<br>Husband / partner disapproved ....4<br>Wanted more effective method........5<br>No method available ....6<br>Health concerns ....7<br>Fear of side effects ....8<br>Lack of access / too far ....9<br>Costs too much ....10<br>Inconvenient to use ....11<br>Fatalistic ....12<br>Difficult to get pregnant / menopausal...................................13<br>Interferes with body's processes ...14	009a=1 AND 306a=1 AND 302a≠1

		Other ....15<br>Don't know ....-88<br>No response.................................-99	
311a	**You first started using [CURRENT/MOST RECENT METHOD] in [DATE FROM 310a or 310c]. Where did you or your partner get it at that time?**<br><br>*Scroll to bottom to see all choices.*	Public sector<br>Govt. Hospital/polyclinic............11<br>Govt. Health center...................12<br>Govt. Health post......................13<br>CHPS.......................................13<br>Public Family planning clinic.....14<br>Mobile clinic..............................15<br>Fieldworker/outreach/peer educator..16<br><br>Private medical sector<br>Private hospital/clinic ....21<br>Private doctor............................22<br>Pharmacy.................................23<br>Chemical/drug store..................24<br>Private FP/PPAG clinic.............25<br>Maternity home.........................26<br><br>Other source<br>NGO...30<br>Shop/market..............................31<br>Church......................................32<br>Community volunteer................33<br>Friend / relative.........................34<br>Herbal Clinic.............................35<br><br>Other ....96<br>Don't know…………………………-88<br>No Response…………………….. -99	(302a=1 & CALC_ CM ≠14, 30, 31, 32)<br><br>OR<br><br>(306a=1 & 306b ≠ 14, 30, 31, 32, 39)
311b	**Where did you learn how to use [RHYTHM/LACTATIONAL AMENORRHEA METHOD]?**<br><br>*Scroll to bottom to see all choices.*	Public sector<br>Govt. Hospital/polyclinic............11<br>Govt. Health center...................12<br>Govt. Health post......................13<br>CHPS.......................................13<br>Public Family planning clinic.....14<br>Mobile clinic..............................15<br>Fieldworker/outreach/peer educator...............................16<br><br>Private medical sector<br>Private hospital/clinic ....21<br>Private doctor............................22<br>Pharmacy.................................23<br>Chemical/drug store..................24<br>Private FP/PPAG clinic.............25<br>Maternity home.........................26<br><br>Other source<br>NGO...30<br>Shop/market..............................31<br>Church......................................32	(302a=1 & CALC_ CM =14, or 30)<br><br>OR<br><br>(306a=1 & 306b = 14 or 30)

		Community volunteer ....33<br>Friend / relative ....34<br><br>Other ....96<br>Don't know…………………………-88<br>No Response…………………….. -99	
312a	**When you obtained your [MOST RECENT / CURRENT METHOD], were you told by the provider about side effects or problems you might have with a method to delay or avoid getting pregnant?**	Yes ....1<br>No...0<br>No response.................................-99	CALC _CM and/or 306b ≠ 14,30,31 ,32, 39
312b	**Were you told what to do if you experienced side effects or problems?**	Yes ....1<br>No...0<br>No response.................................-99	311a = 1
313	**At that time, were you told by a family planning provider about methods of family planning other than the [MOST RECENT/CURRENT METHOD] that you could use?**	Yes ....1<br>No...0<br>No response.................................-99	302a=1 or 306a=1 and CALC _CM and/or 306b !=31,32 ,39
314a	**During that visit, did you obtain the method you wanted to delay or avoid getting pregnant?**	Yes ....1<br>No...0<br>No response.................................-99	302a=1 or 306a=1 and CALC _CM and/or 306b !=14, 30, 31, 32,39
314b	**When you began using [LAM/Rhythm] was this the method you wanted to use to delay or avoid getting pregnant?**	Yes ....1<br>No...0<br>No response.................................-99	CALC _CM and/or 306b = 14 or 30
314c	**Why didn't you obtain the method you wanted?**	Method out of stock that day............1<br>Method not available at all ....2<br>Provider not trained to provide the method ....3<br>Provider recommended a different method ....4<br>Not eligible for method ....5<br>Decided not to adopt a method........6<br>Too costly...7<br>Other ....8<br>No response.................................-99	314a or 314b = 0

315a	**During that visit, who made the final decision about what method you got?**	You alone ....1<br>Provider...2<br>Partner ....3<br>You and provider.............................4<br>You and partner ....5<br>Other ....6<br>No response.................................-99	302a=1 or 306a=1 and CALC _CM and/or 306b !=31,32 ,39
315b	**Who made the final decision to use [LAM/Rhythm]?**	You alone ....1<br>Provider...2<br>Partner ....3<br>You and provider.............................4<br>You and partner ....5<br>Other ....6<br>No response.................................-99	CALC _CM and/or 306b = 14 or 30
316	**Would you return to this provider?**<br><br>**Provider: [Type of Provider from FQ30]**	Yes ....1<br>No..0<br>No response.................................-99	310 ≠ 34 or 96
317	**Would you refer your relative or friend to this provider / facility?**	Yes ....1<br>No..0<br>No response.................................-99	310 ≠ 34 or -96
318a	**In the last 12 months, have you paid any fees for family planning services (including the most current method)?**	Yes ....1<br>No..0	302a or 306a = 1
318b	**How much did you pay?**<br>*Enter all prices in Ghana Cedis. Enter -88 if respondent does not know, -99 for no response.*	Fee [ ]	319 = 1
319	**Have you ever done anything or tried in any way to delay or avoid getting pregnant?**	Yes ....1<br>No..0<br>No response.................................-99	306a ≠ 1
320	**How old were you when you first used a method to delay or avoid getting pregnant?**<br>**The respondent said she was [age from 102] years old at her last birthday.**<br>*Enter the age in years.*<br>*Enter -88 if respondent does not know.*<br>*Enter -99 if there is no response.*<br>*Cannot be younger than 9.*	Age [ ]	302a=1 OR 306a=1 OR 320=1
321	**How many living children did you have at that time, if any?**<br>**Note: the respondent said that she gave birth [number of live births] times in 204.**<br>*Enter -99 for no response*	Number [ ]	Age in 321>0 AND children _born>0
322	**Which method did you first use to delay or avoid**	Female sterilization ....1<br>Male sterilization 2	

	**getting pregnant?**<br>*Do not read the method choices. Be sure to scroll to bottom to see all choices.*	Implant ....3<br>IUD ....4<br>Injectables - 3 months....5<br>Injectables – 1 month....6<br>Pill....7<br>Emergency Contraception ....8<br>Male Condom....9<br>Female Condom ....10<br>Diaphragm....11<br>Foam/Jelly....12<br>Std. Days/Cycle beads....13<br>LAM....14<br>N_tablet....15<br>Rhythm method....30<br>Withdrawal ....31<br>Washing ....32<br>Other traditional methods....39<br>No response…..-99	
323a	**You said that you do not want any / anymore children and that you are not using a method to avoid pregnancy.**<br>**Can you tell me the reason why you are not using a method to prevent pregnancy?**<br>**PROBE: Any other reason?**<br>*RECORD ALL REASONS MENTIONED.*<br>*Cannot select "Do Not Know" or "No response" with other options.*<br>*Cannot select "Not married" if 104 is "Yes, currently married".*<br>*Scroll to the bottom to see all choices.*	Not married ....1/0<br>Infrequent sex / not having sex....1/0<br>Husband away for multiple days....1/0<br>Menopausal/Hysterectomy ....1/0<br>Subfecund / infecund ....1/0<br>Not menstruated since last birth ....1/0<br>Breastfeeding....1/0<br>Up to God / fatalistic....1/0<br>Respondent opposed....1/0<br>Husband / partner opposed ....1/0<br>Others opposed ....1/0<br>Religious prohibition....1/0<br>Knows no method ....1/0<br>Knows no source ....1/0<br>Fear of side effects ....1/0<br>Health concerns....1/0<br>Lack of access / too far....1/0<br>Costs too much....1/0<br>Preferred method not available....1/0<br>No method available ....1/0<br>Inconvenient to use....1/0<br>Interferes with body's processes ....1/0<br>Other ....1/0<br>Don't know ....-88<br>No response....-99	302a = 0<br>AND<br>(212a or 212b > 2 years)
323b	**Would you say that not using contraception is mainly your decision, mainly your husband/partner's decision or did you both decide together?**	Mainly respondent....1<br>Mainly husband/partner ....2<br>Joint Decision....3<br>Other ....96<br>No response....-99	009a=1<br>AND<br>302a = 0
324	**In the last 12 months, were you visited by a community health worker who talked to you about family planning?**	Yes ....1<br>No....0<br>No response....-99	009a = 1

325a	**In the last 12 months, have you visited a health facility or camp for care for yourself or your children?**<br><br>*For any health services*	Yes ....1<br>No..0<br>No response.............................-99	009a = 1
325b	**Did any staff member at the health facility speak to you about family planning methods?**	Yes ....1<br>No..0<br>No response.............................-99	325a = 1

		YES	NO	NR	
326	**In the last few months have you:**				009a = 1
	**a. Heard about family planning on the radio?**	1	0	-99	
	**b. Seen anything about family planning on the television?**	1	0	-99	
	**c. Read about family planning in a newspaper or magazine?**	1	1	-99	
	**d. Received a voice or text message about family planning on a mobile phone?**	1	0	-99	

Section 4. Sexual Activity

CHECK FOR THE PRESENCE OF OTHERS. BEFORE CONTINUING, MAKE EVERY EFFORT TO ENSURE PRIVACY.

401a	**How old were you when you first had sexual intercourse?**<br><br>***The respondent said she was [age from 102] years old at her last birthday.***<br><br>***[She has had x live births.]***<br><br>*Enter the age in years.*<br><br>*Enter -77 if she never had sex.*<br>*Enter -88 if respondent does not know.*<br>*Enter -99 for no response.*	Age ☐	009a = 1
401b	**You have entered that the respondent was X years old when she first had sexual intercourse. Is this what she said?**<br><br>*Go back and correct FQ40a1 if it is not correct.*	Yes ....1<br>No..0	401a ≥ 0 AND 401a < 10 years and ≠ -77, -88 or -99
402	**When was the last time you had sexual intercourse?**<br><br>*If less than 12 months ago, answer must be recorded in months, weeks, or days.*<br><br>*Enter 0 days for today.*<br><br>*You will enter a number for X on the next screen.*	______ days ago<br>______ weeks ago<br>______ months ago<br>______ years ago	401a ≠ -77

Section 6- Menstrual Hygiene

Now I'm going to ask you about menstrual hygiene management. This includes the use of absorbent materials; access to a private, clean, safe space; washing as required; and a place to dispose used materials.

601	**The last time you had your period, where are all the places where you changed, washed, dried, or disposed of used sanitary materials?**	[Main sanitation facility from HQ]..1/0<br>Other household sanitation facility 1/0<br>Sanitation facilities at school.........1/0	009a = 1

	PROBE: Anywhere else?<br>*Do not read options aloud. Select all that apply.*	Sanitation facilities at work............1/0<br>Other public sanitation facility ....1/0<br>Sleeping area/bedroom.................1/0<br>Backyard ....1/0<br>No facility/Bush/Field ....1/0<br>Other ....1/0<br>No response.................................-99	
602	**Where do you most often change your used pads, cloths, or other sanitary materials?**<br>*[ODK will only display the options selected in FQ503]*	[Main sanitation facility from HQ ...1/0<br>Other household sanitation facility 1/0<br>Sanitation facilities at school.........1/0<br>Sanitation facilities at work............1/0<br>Other public sanitation facility ....1/0<br>Sleeping area/bedroom.................1/0<br>Backyard ....1/0<br>No facility/Bush/Field ....1/0<br>Other ....1/0<br>No response.................................-99	More than 1 option selected in 601
602b	**While managing your menstrual hygiene, was this place:**<br>**Main place: [Selection from 503 or 504]**<br>*Read each option aloud and select if yes.*	Clean?..1/0<br>Private?..1/0<br>Safe?..1/0<br>Able to be locked? ....1/0<br>Supplied with clean water? ....1/0<br>Supplied with soap?......................1/0<br>None of the above.........................-77<br>No response.................................-99	503≠-99 nor null and 504≠-99 nor null
603	**During your last menstrual period, what did you use to collect or absorb your menstrual blood?**<br>PROBE: Anything else?<br>*Do not read options aloud. Select all that apply.*	Reusable materials<br>Cloth/Pieces of fabric .... .1/0<br>Cotton wool .... .1/0<br>Sanitary pads .... .1/0<br>Foam (from a mattress or other material) .... .1/0<br>Other .... .1/0<br>Disposable materials<br>Tampons .... .1/0<br>Toilet paper .... .1/0<br>Paper (newspaper, pages from books) .... .1/0<br>Natural materials (mud, dung, leaves).................................. .1/0<br>No materials used .... .1/0<br>No response.............................. -99	009a = 1
604	**Did you wash and reuse pads, cloths, or other sanitary materials during your last menstrual period?**	Yes ....1<br>No...0<br>No response.................................-99	605=Reusable materials
604a	**During your last menstrual period, were the sanitary materials that you washed and reused completely dried before each reuse?**	Yes ....1<br>No...0<br>No response.................................-99	604=1

605	**You mentioned that you used [RESPONSES FROM FQ505] during your last menstrual period. Where did you dispose of these materials after use?**<br><br>PROBE: Anywhere else?<br><br>*Do not read options aloud. Select all that apply.*	Flush toilet....................................1/0<br>Latrine ....1/0<br>Waste bin/Trash bag.....................1/0<br>Burning..1/0<br>Bush/Field ....1/0<br>Other ....1/0<br>No response..................................-99	605=Disposable materials OR 604=0
606	**Is there anything else that would help you manage your menstrual period that you do not usually have?**<br><br>PROBE: Anything else?<br><br>*Could include resources, materials, changes to your environment, etc. Do not read options aloud.*	I have all I need.............................1/0<br>Clean water ....1/0<br>Soap...1/0<br>Clean absorbent materials............1/0<br>A private place ....1/0<br>A place where I feel safe...............1/0<br>More knowledge............................1/0<br>A place to buy clean absorbent materials.......................................1/0<br>A place to dry used materials ....1/0<br>A place to dispose used materials 1/0<br>Money ....1/0<br>Pain medication ....1/0<br>Other ....1/0<br>No response..................................-99	009a = 1
**Primary Health Care Module: Section 1**			
PHC 101a	**Have you visited a health facility for yourself or your child in the last six months?**	Yes...... ....1<br>No ....0<br>No response...-99	PHC 001a=1
PHC 101b	**Why not?**<br>*Do not read options aloud. Select all that are mentioned.*	Not sick/did not need care ....1/0<br>Facility is too far away .... 1/0<br>Too expensive.......................................1/0<br>Too difficult to get to............................1/0<br>Distrust of provider/facility ....1/0<br>Negative prior experience ....1/0<br>Lack of privacy or confidentiality ....1/0<br>Did not know where to go ....1/0<br>CHW provided needed services in house/community ....1/0<br>Other..1/0<br>Do not know .... -88<br>No response...-99	PHC 101a=0
PHC_ 101c	*You selected "other" on the previous screen. Please note down the response that was given here.*	Specify [ ]	"Other" selected in PHC 101b
PHC 102a	**During your most recent visit to the health facility, for whom were you seeking care?**<br><br>*Do not read out loud.*	Yourself..1<br>Your child..2<br>Another family member ....3<br>Other...4<br>No response..-99	PHC 101a=1

PHC 102b	*You selected "other" on the previous screen. Please note down the response that was given here.*	Specify [ ]	PHC 102=4
PHC 103a	**For what reasons were you seeking care?**<br>*Do not read out loud. Select all that apply*	Family planning....................................1/0<br>Maternity services ....1/0<br>Vaccination..1/0<br>Malaria/fever ....1/0<br>Feel sick (undifferentiated symptoms) .1/0<br>CHW told me to go ....1/0<br>Snake bite ....1/0<br>Injury ....1/0<br>Blood pressure ....1/0<br>Diabetes ....1/0HIV testing/treatment ....1/0<br>Worried about a new symptom or feeling ....1/0<br>Other ....1/0<br>No response ....-99	PHC 101a=1
PHC 103b	*You selected "other" on the previous screen. Please note down the response that was given here.*	Specify: [ ]	"Other" selected in PHC 103
PHC 104	**What type of health facility did you visit?**<br><br>*This also refers to the most recent visit the respondent made. Do not read out loud. Select the one that best applies.*	Public sector<br>Govt. Hospital/polyclinic............11<br>Govt. Health center ....12<br>Govt. Health post ....13<br>CHPS ....13<br>Family planning clinic................14<br>Mobile clinic...............................15<br><br>Private medical sector<br>Private hospital/clinic ....21<br>Private doctor ....22<br>Pharmacy ....23<br>Chemical/drug store ....24<br>FP/PPAG clinic ....25<br>Maternity home ....26<br><br>Other source<br>NGO ....30<br>Herbal Clinic.................................35<br><br>Other ....96<br>Don't know…………………………-88<br>No Response…………………….. -99	PHC 101a=1
PHC 104b	**What was the name of the facility you visited?**	[ODK will display a list of facilities based on the location selected.]<br><br>None of the above............................... -77<br>Do not know .... -88<br>No response...-99	PHC101 a=1 AND PHC 104 ≠ -99

PHC 105a	**Please tell me the most important things that influence your decision about where to seek health services.**<br><br>**PROBE: Anything else?**<br><br>*Do not read out loud. Select all answers that apply.*	Waiting time to see doctor....................1/0<br>Distance to facility.................................1/0<br>Cleanliness of facility............................1/0<br>Being treated with respect ....1/0<br>Competence/ knowledge of provider....1/0<br>Confidentiality/privacy.........................1/0<br>Availability/supply of medicines..........1/0<br>Cost of treatment ....1/0<br>Being able to choose health care provider ....1/0<br>Personally know health care provider ..1/0<br>Prefer traditional healers.......................1/0<br>Other, specify ....1/0<br>No response..-99	PHC 001a=1
	You selected "other" on a previous screen. Please note down the response that was given here.	Specify:	"Other" selected in PHC 106a
105b	**You mentioned that: [ANSWERS SELECTED IN 105a] are important to you in deciding where to seek health care services. Can you tell me which of these things is the most important to you?**	Waiting time to see doctor.......................1<br>Distance to facility..................................2<br>Cleanliness of facility..............................3<br>Being treated with respect ....4<br>competence/ knowledge of provider ....5<br>Confidentiality/privacy............................6<br>Availability/supply of medicines............7<br>Cost of treatment ....8<br>Being able to choose health care provider9<br>Personally know health care provider ...10<br>Prefer traditional healers........................11<br>Other, specify ....12<br>No response .... -99	More than one option selected in 105a
PHC 106	**If you or your child were to get sick tomorrow, how easy or difficult would it be for you to get the care you need?**<br><br>*Read the question and answer choices out loud and ask the respondent to pick the best answer.*	Very difficult ....1<br>Difficult ....2<br>Easy ....3<br>Very easy...4<br>No response..-99	PHC 001a=1
PHC 107	**When you go to your primary care site, are you taken care of by the same provider each time?**<br><br>*Read the question and answer choices out loud and ask the respondent to pick the best answer.*	Always...1<br>Frequently..2<br>Rarely ....3<br>Never ....4<br>Do not know .... -88<br>No response..-99	PHC 001a=1

Thinking about the last time you visited your local health facility for yourself or your child for any reason, please answer the following questions.

PHC 108a	**How long did you wait before being seen by your provider?**<br><br>*Record in unit respondent provides.*	X minutes ....1<br>X hours ....2<br>Gave up without seeing provider....................3<br>Do not know .... -88<br>No response....................-99<br><br># Minutes [ ]<br># Hours [ ]	PHC 101a-=1
PHC 108b	**How would you rate this waiting time?**<br>*Read the question and answer choices out loud and ask the respondent to pick the best answer.*	Unbearable....................1<br>Very long....................2<br>Long....................3<br>A little long....................4<br>Fine....................5<br>No response....................-99	PHC 109a ≠ -88, -99 AND 101a=1
PHC 109	**How would you rate the cleanliness of the rooms inside the facility, including toilets?**<br>*Read the question and answer choices out loud and ask the respondent to pick the best answer.*	Excellent....................1<br>Very good....................2<br>Good....................3<br>Fair....................4<br>Poor....................5<br>No response....................-99	PHC 101a=1
PHC 110	**Please tell me if you agree or disagree with the following statement: You trust the skills and abilities of the health workers at this facility.**<br>*Read the question and answer choices out loud and ask the respondent to pick the best answer.*	Strongly agree....................1<br>Agree....................2<br>Disagree....................3<br>Strongly disagree....................4<br>No response....................-99	PHC 101a=1
PHC 111	**How easy or difficult was it to understand the information your provider gave to you?**<br>*Read the question and answer choices out loud and ask the respondent to pick the best answer.*	Very difficult....................1<br>Difficult....................2<br>Easy....................3<br>Very easy....................4<br>No response....................-99	PHC 101a=1
PHC 112	**How easy or difficult was it for you to follow the provider's advice?**<br>*Read the question and answer choices out loud and ask the respondent to pick the best answer.*	Very difficult....................1<br>Difficult....................2<br>Easy....................3<br>Very easy....................4<br>No response....................-99	PHC 101a=1
PHC 113	**How would you rate the level of respect shown to you by the providers and staff at this facility during this visit?**<br>*Read the question and answer choices out loud and ask the respondent to pick the best answer.*	Excellent....................1<br>Very Good....................2<br>Good....................3<br>Fair....................4<br>Poor....................5<br>No response....................-99	PHC 101a=1
PHC 114	**Overall, taking everything into account, how would you rate the quality of care you received at this facility?**<br>*Read the question and answer choices out loud and ask the respondent to pick the best answer.*	Excellent....................1<br>Very Good....................2<br>Good....................3<br>Fair....................4<br>Poor....................5<br>No response....................-99	PHC 101a=1

PHC 115	**How likely are you to return or bring your children to this facility for health care in the future?**<br>*Read the question and answer choices out loud and ask the respondent to pick the best answer.*	Extremely likely ....1<br>Likely..2<br>Unlikely ....3<br>Extremely unlikely ....4<br>Do not know .... -88<br>No response..-99	PHC 101a=1
PHC 116	**Were your services covered by insurance?**	Yes...1<br>No ....0<br>No response..-99	PHC 101a=1
PHC 117	**Which insurance program covered these services?**<br>*Do not read out loud. Record all that are mentioned.*	National/District Health Insurance ....1/0<br>Health insurance through employer......1/0<br>Mutual health organization/ community based insurance....................................1/0<br>Other privately purchased commercial health insurance................................... 1/0	PHC 117=1
PHC 118a	**Was the cost to you lower because you had insurance?**	Yes...1<br>No ....0<br>Do not know .... -88<br>No response .... -99	PHC 117=1
PHC 118b	**How much did you have to pay out of pocket?**<br>*Record amount in GHC*<br>*Enter -99 for no response*	Amount	PHC 101a=1
PHC 119	**How easy or difficult was it for you to pay for this visit?**<br>*Read the question and answer choices out loud and ask the respondent to pick the best answer.*	Very difficult ....1<br>Difficult ....2<br>Easy ....3<br>Very easy...4<br>No response..-99	PHC 101a=1
PHC 120	**Did you have to borrow money or sell something to afford the costs of this visit, including all costs such as transportation and lost wages?**	Yes...1<br>No ....0<br>No response..-99	PHC 101a=1
**END OF SURVEY**<br>**Thank the respondent for her time**<br>*The respondent is finished, but there are still 4 more questions for you to complete outside the home.*			
096	**Location**<br>*Take a GPS point near the entrance to the household. Record location when the accuracy is smaller than 6m.*<br>*GPS coordinates can only be collected when outside.*	**LOCATION**	Always
097	**How many times have you visited this household to interview this female respondent?**	1st time... 1<br>2nd time .... 2<br>3rd time 3	Always
098	**In what language was this interview conducted?**	English .... 1<br>Akan .... 2<br>Ga... 3<br>Ewe .... 4<br>Nzema... 5<br>Dagbani... 6<br>Other 96	009a=1
099	**Questionnaire result**	Completed.. 1	Always

	Record the result of the Female Questionnaire	Not at home....................................... 2<br>Postponed... 3<br>Refused... 4<br>Partly completed .... 5<br>Incapacitated..................................... 6	
