# Supplementary material for: Facility management associated with improved primary health care outcomes in Ghana
Source: PLoS One. 2019 Jul 2;14(7):e0218662. doi: 10.1371/journal.pone.0218662 (PMC6605853; doi:10.1371/journal.pone.0218662)
Supplement: S5 File — Details of the 27 indicators which comprise the management index. (PDF) [file pone.0218662.s005.pdf]

Supplementary Information 5. Management component indicators based on the World Management Survey framework

	Questions to facility head:			
Target		0		1
	**a. Has annual budget for running costs:** Does your facility have one comprehensive annual budget for running costs? By running costs, I mean all of the costs of operating this facility, including paying staff, building maintenance, and purchasing of supplies, equipment, medicines, and utilities.	Do not know/ No		Yes
	**b. Facility accountable for certain population:** Is your facility accountable for the health outcomes of a group of people, even if they never attend services at your health facility?	Do not know/ No		Yes
	**c. Measure coverage of key population indicators:** Are you required to measure coverage of key population indicators, such as immunization coverage?	Do not know/ No		Yes
	**Target score:**	**_______ /3 = __________**		
Operations		**0**	**0.5**	**1**
	**a. Operating hours:** Is this facility open every day? [Original question: How many days each week is the facility routinely open?]	No		Yes
	**b. User fees displayed:** [Observed] Are any user fees/charges displayed at the facility? Probe: May I see where they are displayed?	No		Yes
	**c. Healthcare worker present 24 hours a day:** Is there a healthcare worker present at the facility at all times or officially on call for the facility at all times (24 hours a day) for emergencies?	No		Yes
	**d. Formal training**: Have you ever received any formal training in the management of a health facility?	No		Yes
	**e. Handwashing station with soap and water:** May I see a nearby handwashing facility that is used by staff?	None	Soap or water	Soap and water
	**f. Time spent for managerial activities yesterday:**<br>Determined from two questions: How many total hours did you work [yesterday] at this facility? During this day, how much time did you devote to each of these activities:<br>1. Overseeing patient flow (e.g., patient admissions, triage, transfers, and discharges);<br>2. Supervising medical staff (e.g., meeting with staff, providing feedback, checking absenteeism.);<br>3. Managing operational budgets (e.g., tracking revenue, submitting claims, paying bills);<br>4. Verifying/Ensuring availability of drugs and equipment (e.g., taking inventory, placing orders, etc.); and<br>5. Managing relationships with staff, community, facility committee, donors, and government	**Proportion of average managerial time spent:**		
	**Operations score:**	**_______ /6 = _________**		
Human resources		**0**		**1**
	**a. Criteria to evaluate staff performance:** Do you have a set of established criteria your facility uses to evaluate staff performance?	Do not know/ No		Yes
	**b. Supervisors review staff performance in last year:** In the past 12 months, have supervisors at your facility held individual meetings with staff to review their performance?	Do not know/ No		Yes
	**c. Offer trainings to staffs:** Are staff in the facility offered trainings to improve their skills?	Do not know/ No		Yes
	**d. Main method of supervision:** What is the main method of supervision in place in your facility?	**Score:**		

	1. Formal supervision process with regular pre-arranged supervision meetings<br>2. Supervision is only available if requested by staff<br>3. Supervision is supportive and continuous<br>4. Supervision consists of negative feedback when performance is poor<br>5. None - no method of supervision OR No response [Note: Original has "Other" as an option, but no one responded "Other"]<br>**Score conversion: 1=0.66 | 2=0.33 | 3=1 | 4=0.33 | 5=0**		
	**Human resource score:**	**_______ /4 = __________**	
Monitoring		**0**	**1**
	**a. Report new outbreaks:** Do you have a mechanism to collect and report new disease outbreaks?	Do not know/No	Yes
	**b. Track common conditions:** Does this facility regularly receive reports tracking the most common health conditions and outcomes in the community?	Do not know/ No	Yes
	**c. Any results collected using any tool:** Are the results of the health conditions and outcomes collected and shared with facility staff through any means? Tools can include: displayed in the facility (chalkboard, poster, noticeboard), staff meetings, on individual basis as requested, other, not collected or shared	Do not know/ No	Yes (any tool)
	**d. Reports client opinion:** Is there a procedure for reviewing or reporting on clients' opinions?	Do not know/ No	Yes
	**e. Facility has quality improvement activities:** Does this facility conduct any quality improvement activities?	Do not know/ No	Yes
	**f. Discussed routine service statistics with staff:** In the past 12 months, have there been any meetings where routinely collected service statistics or clinical audit data are discussed with staff?	Do not know/ No	Yes
	**g. Books for tracking:** Does your facility maintain books to track revenue and expenditures? Probe: May I see the books?	Do not know/ No	Yes
	**h. Data for service delivery:** On a scale of 1 (strongly disagree) to 5 (strongly agree), How much do you agree or disagree with the following statement: The use of data to monitor and improve service delivery is highly valued in this facility.<br>**Score conversion: 1 = 0 | 2 = 0.25 | 3 = 0.50 | 4 = 0.75 | 5 = 1**	**Rate:**	
	**Monitoring score:**	**_______ /8 = __________**	
Community engagement		**0**	**1**
	**a. Community advisory board meetings:** Does this facility have a community advisory board or community management committee that meets regularly?	Do not know / No	Yes
	**b. Made any change as a result of client opinion:** In the past 6 months have any changes been made in the program as a result of client opinion?	Do not know / No	Yes
	**c. Facility collects client opinion using any tool:** Do you collect information about clients' opinion in any of the following ways? 1. suggestion box, 2. client survey form, 3. structured interviews with clients, 4. official meetings with community leaders, 5. informal discussion with client/community meeting, 6. other	No	Yes (any tool)
	**d. Regular community member in staff meeting:** Is there a community member who regularly attends staff meetings?		
	**e. Shared performance with the community:** In the past 12 months, has this facility shared information on its performance with the community it serves?		

	**f. Patients opinions drive change:** On a scale of 1 (strongly disagree) to 5 (strongly agree), how much do you think that patients' opinions about their experiences at your facility drive change or improvement efforts?<br>**Score conversion: 1 = 0 | 2 = 0.25 | 3 = 0.50 | 4 = 0.75 | 5 = 1**	**Rate:**	
	**Community engagement score**:	**_______ /6 = __________**	
	**TOTAL MANAGEMENT SCORE:**		
