# Supplementary material for: Facility management associated with improved primary health care outcomes in Ghana
Source: PLoS One. 2019 Jul 2;14(7):e0218662. doi: 10.1371/journal.pone.0218662 (PMC6605853; doi:10.1371/journal.pone.0218662)
Supplement: S6 File — List of essential drugs assessed by facility type. (PDF) [file pone.0218662.s006.pdf]

Supplementary Information 6. Essential drugs assessed

Essential drugs	Hospitals	Health centers with doctor	Health centers without a doctor	CHPS
1. Oxytocin	√	√	√	√
2. Misoprostol (cap/tab)	√	√	√	
3. Sodium chloride (saline solution)/ (injection solution)	√	√	√	√
4. Azithromycin (cap/tab or oral liquid)	√			
5. Calcium gluconate (injectable)	√			
6. Magnesium sulfate	√	√	√	√
7. Ampicillin powder (for injection)	√	√	√	√
8. Betamethasone or Dexamethansone (injectable)	√			
9. Gentamicin (injectable)	√	√	√	
10. Nifedipine (cap/tab)	√	√		
11. Metronidazole (injectable)	√	√	√	√
12. Iron supplements (cap/tab)	√	√	√	√
13. Folic acid supplements (cap/tab)	√	√		√
14. Amoxicillin (syrup/suspension)	√	√	√	√
15. Oral Rehydration Salts (ORS sachets)	√	√	√	√
16. Zinc (tablets)	√	√	√	√
17. Ceftriaxone (injectable)	√			
18. Artemisinin combination therapy (ACT)	√	√	√	√
19. Artusunate (rectal or injectable)	√	√	√	√
20. Benzylpenicillin (powder for injection)	√	√		
21. Vitamin A (capsules)	√	√	√	√
Total:	21	17	14	13

Source: Drug list selected from the Service Delivery Indicators | Health Indicators (Essential Drug List). http://www.sdindicators.org/indicators and matched with Ghana Essential Medicines List, Ministry of Health (GNDP) Ghana. 2010 www.ghndp.org.
