# Supplementary material for: Facility management associated with improved primary health care outcomes in Ghana
Source: PLoS One. 2019 Jul 2;14(7):e0218662. doi: 10.1371/journal.pone.0218662 (PMC6605853; doi:10.1371/journal.pone.0218662)
Supplement: S7 File — List of family planning types assessed by facility type. (PDF) [file pone.0218662.s007.pdf]

Supplementary Information 7. Family planning types assessed

Family planning types	Hospitals and polyclinics	Health centers and clinics	CHPS
Male condom	√	√	√
Female condom	√	√	√
Diaphragm	√	√	√
Spermicides (foam)	√	√	√
Oral contraceptives (pills)	√	√	√
Beads	√	√	√
Implants	√	√	√
Injectable at 1 month	√	√	√
Injectable at 3 months	√	√	√
IUD/IUS	√	√	√
Emergency contraception	√	√	√
Female sterilization (tubal ligation)	√		
Male sterilization (vasectomy)	√		
Lactational amenorrhea method*	√	√	√
Rhythm*	√	√	√
Withdrawal*	√	√	√
Total:	16	14	14

* Only assessed whether these natural family planning methods were counseled, not whether they were provided.
Source: Ghana Health Service. National reproductive health service policy and standards: Third edition. December 2014. Page 12
