# Supplementary material for: Facility management associated with improved primary health care outcomes in Ghana
Source: PLoS One. 2019 Jul 2;14(7):e0218662. doi: 10.1371/journal.pone.0218662 (PMC6605853; doi:10.1371/journal.pone.0218662)
Supplement: S8 File — Figure showing variation in performance of management overall as well as each management domain, by region and facility type. (PDF) [file pone.0218662.s008.pdf]

Supplementary Information 8. Management domains by region and facility type

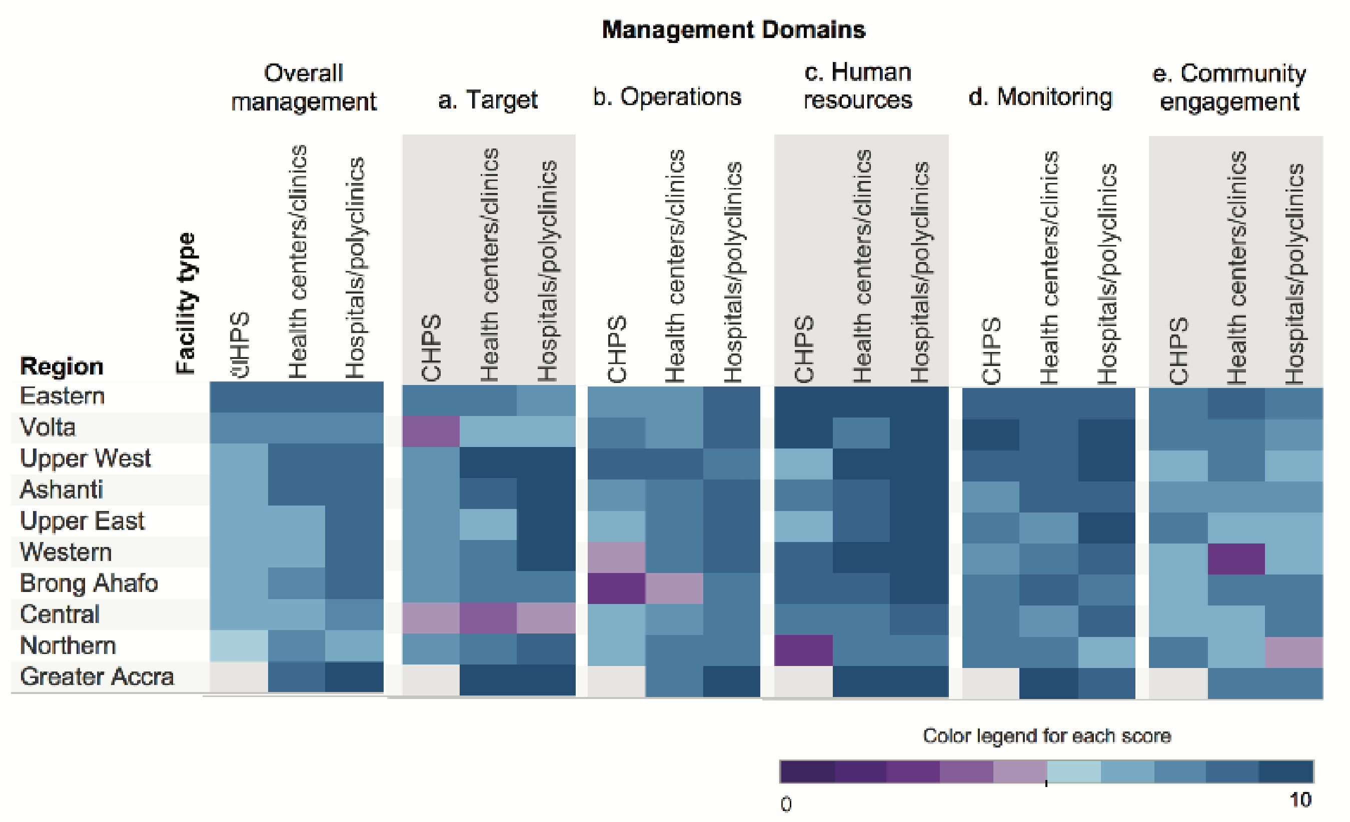

Management scores are from a scale of 0 (lowest, in dark purple) to 1 (highest, in dark blue).
Community-based Health Planning and Services (CHPS) are not included in the sampled facilities in Greater Accra.
