# Supplementary material for: Facility management associated with improved primary health care outcomes in Ghana
Source: PLoS One. 2019 Jul 2;14(7):e0218662. doi: 10.1371/journal.pone.0218662 (PMC6605853; doi:10.1371/journal.pone.0218662)

Supplementary Information 9. Process outcomes by region

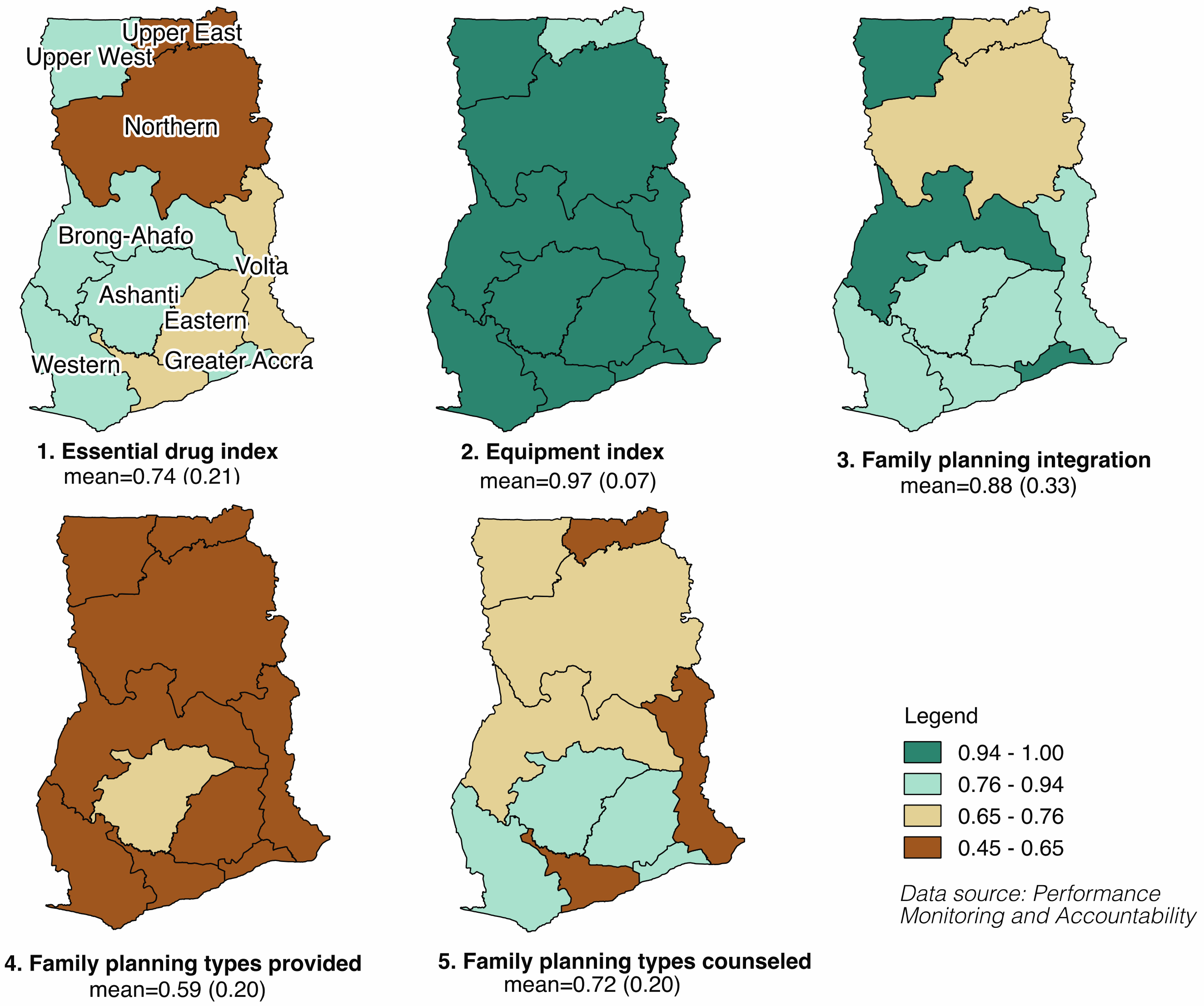

Supplement: S9 File — Maps showing regional variation across Ghana in process outcomes. (PDF) [file pone.0218662.s009.pdf]
